# Effective Molecular Dynamics from Neural Network-Based Structure Prediction Models

**DOI:** 10.1021/acs.jctc.2c01027

**Published:** 2023-03-24

**Authors:** Alexander Jussupow, Ville R. I. Kaila

**Affiliations:** Department of Biochemistry and Biophysics, Stockholm University, 10691 Stockholm, Sweden

## Abstract

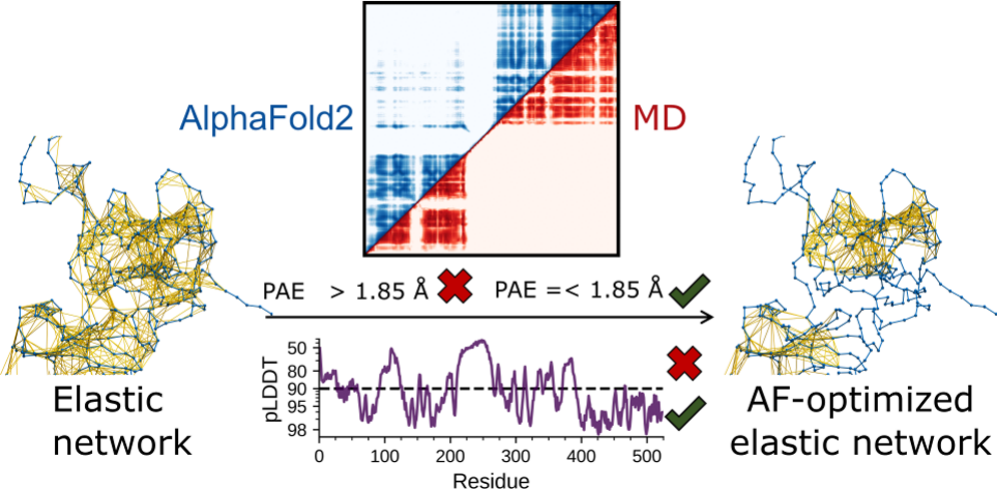

Recent breakthroughs in neural network-based structure prediction methods, such as AlphaFold2 and RoseTTAFold, have dramatically improved the quality of computational protein structure prediction. These models also provide statistical confidence scores that can estimate uncertainties in the predicted structures, but it remains unclear to what extent these scores are related to the intrinsic conformational dynamics of proteins. Here, we compare AlphaFold2 prediction scores with explicit large-scale molecular dynamics simulations of 28 one- and two-domain proteins with varying degrees of flexibility. We demonstrate a strong correlation between the statistical prediction scores and the explicit motion derived from extensive atomistic molecular dynamics simulations and further derive an elastic network model based on the statistical scores of AlphFold2 (AF-ENM), which we benchmark in combination with coarse-grained molecular dynamics simulations. We show that our AF-ENM method reproduces the global protein dynamics with improved accuracy, providing a powerful way to derive effective molecular dynamics using neural network-based structure prediction models.

## Introduction

AlphaFold2^1^ and RoseTTAfold^2^ can infer protein structures based on sequence information with high accuracy. As a result, many projects have emerged that aim to improve the accessibility and usability of these structure prediction tools, most notably the AlphaFold Protein Structure Database,^3^ which currently provides predicted structures for over 200 million proteins,^4^ ColabFold,^5^ which combines fast homology search of MMseqs2^6^ with AlphaFold2 and RoseTTAfold, and derivations to predict large protein complexes.^7,8^

AlphaFold uses the predicted local distance difference test (pLDDT) to estimate the accuracy of the predicted C_α_ positions (on a relative scale of 0–100) with experimental structures,^1,9,10^ but it remains unclear to what extent these measures can be linked to physical properties, such as protein dynamics. While low pLDDT scores have been associated with intrinsically disordered regions (IDRs),^3,10−13^ conditionally folded IDRs, i.e., protein regions that fold in the presence of a binding partner or post-translational modifications, can show high (>70–90) pLDDT scores. In this regard, AlphaFold2 often appears to favor the folded state,^14^ possibly as these are structurally overrepresented in the protein database. Highly dynamic protein conformations can also be identified by analyzing the combined pLDDT score, their solvent accessibility,^15^ and amino acid sequences that usually comprise a high proportion of charged and hydrophobic residues.^14^

AlphaFold2 also uses the predicted aligned error (PAE) as another metric to estimate positional errors for a given residue, *i*, between the predicted and experimental structures that are aligned relative to residue *j* (capped at 31.75 Å),^1,3^ an approach adapted from superposition-based metrics.^16,17^ PAE scores can be represented as a nonsymmetric matrix related to the model confidence for the relative position and orientation of different protein segments. It is thus possible that the PAE scores could be used to determine rigid cores and protein domains.^11,18^ Qualitative comparisons with atomistic molecular dynamics (aMD) simulations seem to confirm these observations.^19^ Recent studies have also explored other approaches to gain information about protein dynamics using the variation in the predicted structures with AlphaFold2,^20^ but a systematic strategy to infer protein dynamics from AlphaFold prediction scores is still lacking.

Here, we compare AlphaFold prediction scores for a set of 28 different single and two-domain proteins against aMD simulations, with the systems chosen to capture proteins with varying degrees of flexibility. Although aMD simulations provide an accurate basis for the structural characterization of the conformational space, the description of highly dynamical proteins, e.g., with intrinsically disordered regions (IDRs) or flexible domains, remains challenging and limited in system size and the timescales needed to capture the major conformational changes.^21,22^ To address this challenge, we propose a framework to convert the AlphaFold scores into elastic network models (ENMs).^23−25^ The core assumption behind an ENM is that the protein dynamics can be adequately described by fluctuation around an initial, native structure and can be used to propagate effective molecular dynamics of the system at a low computational cost.

ENMs are often used in combination with coarse-grained MD (cgMD) simulations. To sample the large-scale conformational dynamics of biomolecules with cgMD models, multiple heavy atoms are grouped into individual interaction centers or beads, reducing the system’s degrees of freedom and allowing for larger computational time- and size-scales. However, these approximations also lead to a reduction in accuracy, particularly for proteins, which is partially accounted for by the restraints from the ENMs.^26^ Among the various coarse-grained approaches, the MARTINI force field^24,27−29^ has proven to be highly versatile due to its balance between chemical specificity and performance.^26,30^ Initially developed for lipids,^27^ the MARTINI model was later extended to include parameters for proteins,^24^ DNA,^31^ RNA,^32^ carbohydrates,^33^ as well as many small molecules.^34^ The latest MARTINI3 version^29^ has expanded available bead types, resulting in more accurate and detailed representations of functional groups, although both former and current models still struggle to properly balance the challenging protein–protein and protein–solvent interactions. In this regard, increased protein–water (P–W) interactions can improve the agreement with experimental data.^35−39^ Due to the introduced approximations, the MARTINI model also encounters difficulties in preserving secondary and tertiary structure elements of proteins, which is why it is often combined with ENMs^23−25^ or Go̅-type models^40,41^ in which the harmonic bonds are substituted by Lennard-Jones interactions.

After establishing a link between protein dynamics by benchmarking data from around 62 μs of aMD simulations and the statistical AlphaFold scores (pLDDT, PAE), we develop here an AlphaFold-optimized ENM (AF-ENM) and show that it can be used in combination with MARTINI cgMD simulations to increasing their usability for dynamical proteins.

## Results

### Correlation between AlphaFold Scores and Protein Dynamics

To probe how the pLDDT and PAE scores correlate with the protein conformational dynamics, we performed aMD simulations of 28 different proteins (Figure 1, Table S1, Figures S1–S28). Each protein was simulated for 2 μs, except the single-domain proteins (protein 24–26), which were simulated for 1 μs. Moreover, we used a 10.5 μs SAXS-reweighted ensemble for the two-domain model of the highly flexible heat shock protein 90 (NTD-MD construct of Hsp90, protein 28) from a previous study,^42^ leading to benchmarking data set of 61.5 μs aMD simulation. The aMD simulations were performed using the a99SB-disp force field, as it accurately captures both folded and disordered protein states.^43^

**Figure 1:**
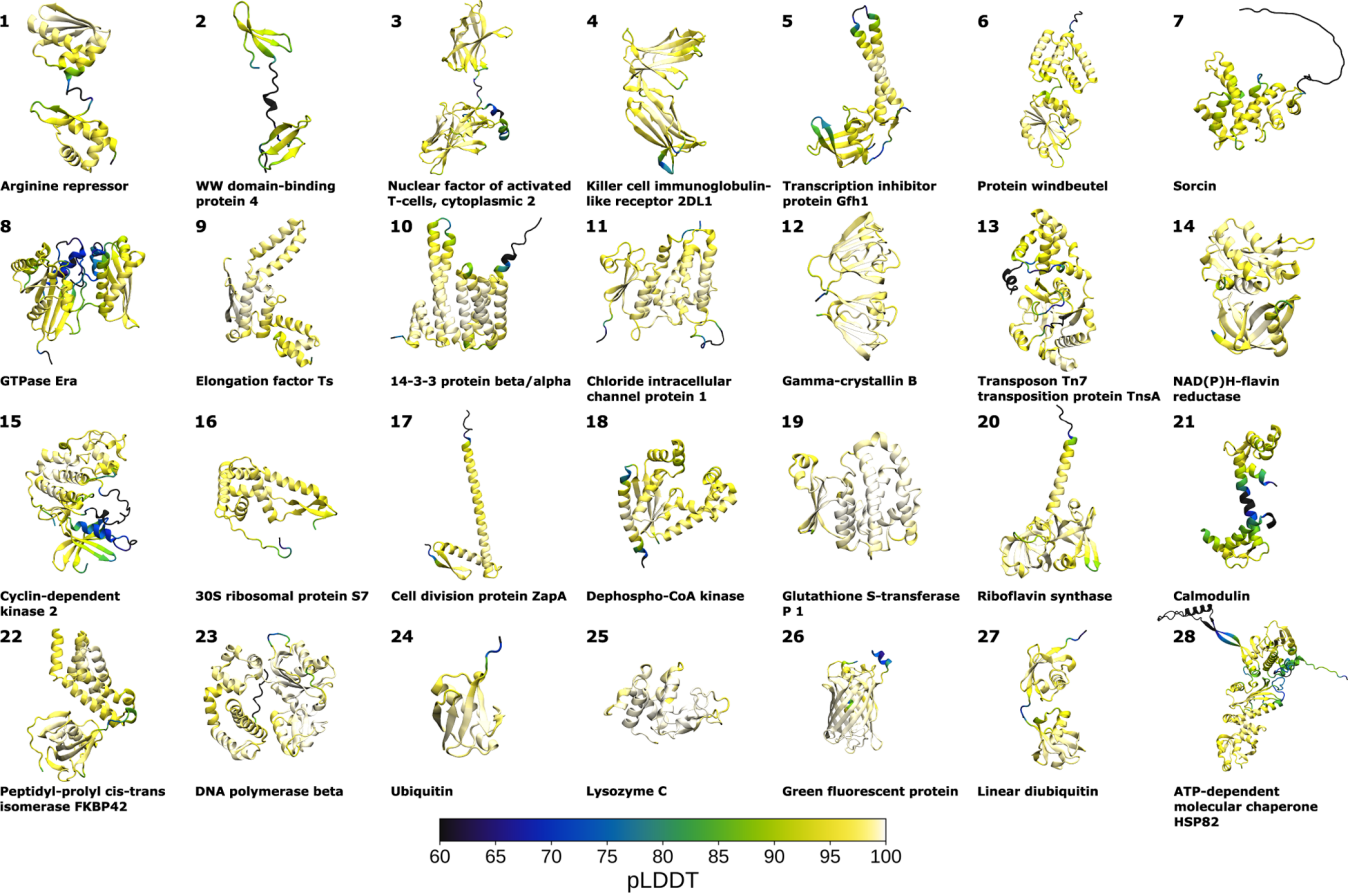
Structure of 28 proteins used to compare statistical scores of AlphaFold with dynamics derived from atomistic MD simulations. The proteins are colored based on their pLDDT scores, with pLDDT scores ≤60 (dark blue) and residues with a pLDDT ∼100 (white).

To evaluate the global protein dynamics, we used the standard deviation of C_α_–C_α_ distance σ_d_ across the cgMD trajectory (with *N* frames)
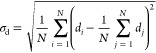
1Combined, the σ_d_s of all distances C_α_–C_α_ can be represented as a matrix similar to the PAE scores. To assess the degree of flexibility of individual residues, we used the average standard deviation of the C_α_-distances to the *n* (nonbonded) nearest amino acids (σ_d,n_)
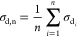
2Unlike the widely used root-mean-square-fluctuation (RMSF) metric, the σ_d,n_ does not depend on structural alignment and is therefore well suited for (highly flexible) multidomain proteins. Overall, groups of residues with higher σ_d,20_ and σ_d_ values can be interpreted as regions with high conformational flexibility.

In Figure S29, we test the correlation between pLDDT and σ_d,n_ scores (as described below) using different *n* values and different linear and nonlinear correlation metrics. In this regard, we used the Pearson correlation coefficient (*R*) as a measure of linear correlation, Spearman’s rank correlation (ρ) to assess the monotonic relationship between two variables, the Kendall rank correlation (τ) to test the ordinal association, as well as mutual information (MI) correlation as a measure of the mutual dependency. To further test σ_d,n_, we also compared correlations using all C_α_ atoms in a given sphere instead of considering only the *n* closest residues (Figure S29b,d). Overall, we find that using a specific number of nearest C_α_ atoms provides a higher correlation relative to a fixed sphere approach. The highest *R* is achieved with *n* = 13, while ρ and τ peak at 46 and MI correlation at 37, with ρ showing the highest values. We find that *n* = 20 gives a reasonable compromise between all correlation scores, with a minimal dependency on the number of atoms, as values ranging from 10 to 70 atoms yield similar results. In the following sections, we will solely refer to the linear correlation *R* as it is the most widely used correlation metric. However, we would note that the ρ values are slightly higher, indicating that the pLDDT vs σ_d,20_ have a monotonic character, which is not fully captured by the linear correlation analysis.

**Figure 2:**
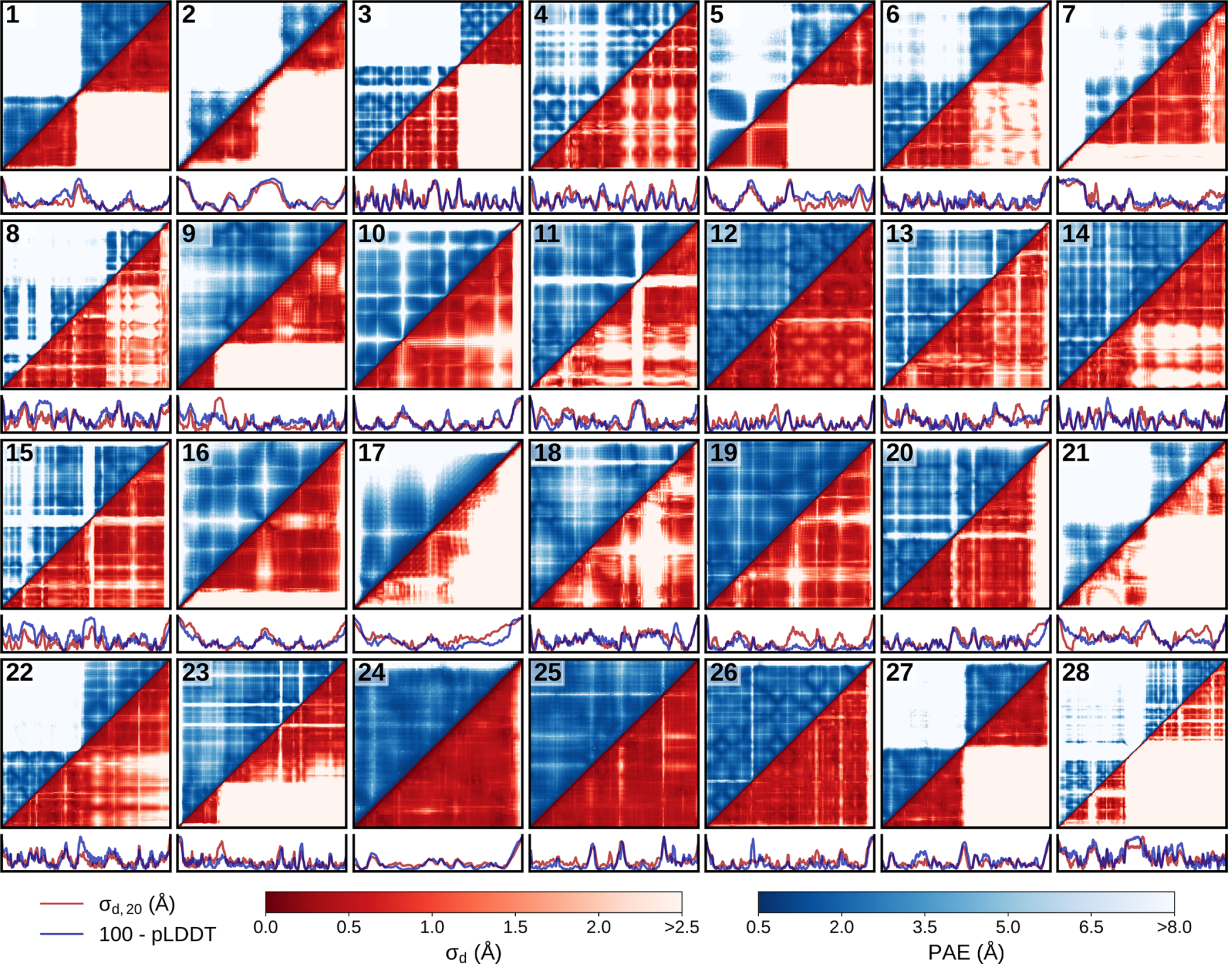
Overview of AlphaFold scores and protein dynamics metrics for all studied systems. A fully symmetric matrix indicates a perfect correlation between the AlphaFold and MD data. The figure is divided into 28 subfigures for each of the proteins shown in Figure 1. The top matrices show a comparison between (symmetrized) PAE matrices (blue) against the standard deviation of all C_α_ distances σ_d_ (red). The PAE scores range between 0.5 and 8.0, while the threshold for σ_d_ is set to <2.5 Å to enable better comparisons between the different systems. The bottom graphs compare the pLDDT scores (blue) and average standard deviation of the Cα-distances to the 20 closest amino acids σ_d,20_ (red). See Figures S1–S28 for a detailed analysis of the individual systems.

A comparison between the PAE (in blue) and σ_d_ (in red) matrices, as well as the pLDDT and σ_d,20_ scores, for the 28 systems, are shown in Figure 2, with a more detailed analysis of the individual proteins in Figures S1–S28. We note that high pLDDT scores correspond to low σ_d,20_ values, with an overall correlation coefficient *R* of 0.65, with a 1 Å increase in the σ_d,20_ corresponding to a 9-unit decrease of the pLDDT score. The overall linear regression across all tested proteins is
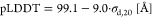
3We observe that the *R*-values for the regressions of individual systems vary between 0.24 and 0.99, with a median value of 0.74 (Table S2). While the slopes vary from −1.3 to −21.7 (median of −10.3), the intercept range is between 94.1 and 106.3 (median of 101.3). Regions with low pLDDT scores generally correspond to high σ_d,20_ values, particularly for disordered proteins regions, flexible loops, and linker regions. However, the relative heights of the peaks do not always align (e.g., Figure S4a). Our analysis thus suggests that the pLDDT scores can be used to identify more flexible regions within a given protein rather than comparing the absolute dynamics between different systems. Based on the regression between the PAE scores and σ_d_ metric across all systems, we find an *R* of 0.53, which is lower than for pLDDT—σ_d,20_. Overall, a σ_d_ increase of 1 Å corresponds to a rise in PAE of 0.7 Å
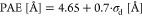
4The *R*-values for individual systems range from 0.25 to 0.92 (median 0.65), with intercepts within the 0.10 to 6.27 range (median 2.4) and the slopes between 0.38 and 4.83 (median 2.1), suggesting that the comparison of PAE values between the different systems have considerable uncertainties but rather good correlation with the explicit dynamics within the same system (Table S3). While the overall correlations are not necessarily high enough for a quantitative assessment of protein dynamics, we later show that a threshold-based approach can reliably differentiate between rigid and flexible regions and be used for constructing ENMs (see below).

Overall, the qualitative consistency of the pLDDT and PAE scores across all studied protein systems is striking. We notice that a PAE score of ∼8 Å appears to set a good threshold to visibly identify highly flexible regions as well as dynamically separated domains, corresponding to a σ_d_ of around 2.5 Å. For example, for protein systems 1, 2, 3, 21, and 27 (arginine repressor, WW domain-binding protein 4, nuclear factor of activated T-cells cytoplasmic 2, calmodulin, and linear diubiquitin), the PAE threshold correctly predicts the high inter-domain flexibility. AlphaFold also appropriately assesses the increased flexibility of N-terminal (system 7 and 16, sorcin and 30S ribosomal) and C-terminal tails (e.g., system 17, 20, 24, and 26 (cell division protein ZapA, riboflavin synthase, ubiquitin, and green fluorescent protein)), as well as dynamic linker regions (e.g., systems 2, 11, and 28 (WW domain-binding protein 4, chloride intracellular channel protein 1, and NTD-MD construct of Hsp90)). Generally, residues with lower pLDDT scores show higher PAE scores for residue pairs, suggesting that the PAE matrix also encodes the local dynamics, e.g., flexible loop regions. This close link between the scores might explain some of the observed discrepancies between PAE scores and the σ_d_ metric in systems 9, 22, and 23 (elongation factor Ts, peptidyl–prolyl *cis*–*trans* isomerase FKBP42, and DNA polymerase β). The differences could be attributable to the over- or underestimation of the conformational dynamics in a small linker section between two domains, with errors that could propagate and lead to significant changes in the PAE scores.

Based on a closer look at our largest and most complicated system within the data set, the NTD-MD construct of Hsp90 (protein 28, Figure S28), we observe an interplay of the abovementioned effects. This system is composed of an N-terminal domain (NTD) and a middle domain (MD), which are connected by a highly charged disordered linker (CL) region.^44−48^ In a previous study, we showed that conformational changes between compact and extended states are enabled by the dissociation of the NTD from the MD.^42^ Moreover, additional conformational dynamics is enabled by the flexible lid region in the NTD, which has well-established open and closed states and a potential unfolding of a β-sheet comprising β-strand 8 from the CL and β-strand 7 from the NTD,^44,48^ with partial unfolding leading to highly extended conformations.^42^ Thus, the Hsp90 NTD-MD model comprises a mixture of rigid domains, IDRs, and conditionally folded states. We indeed observe that the pLDDT scores and the σ_d,20_ metrics for the Hsp90 construct within the individual regions are consistent with the flexibility derived from the aMD simulations. The exception is the β8-strand, where we notice a pLDDT score of >90 for one of the highly dynamic regions. This finding is consistent with the observation that AlphaFold generally predicts high pLDDT values for residues in conditionally folded areas.^14^ The PAE and σ_d_ scores between the NTD and MD domains are indeed significantly higher than those within each domain. For residues in the CL, both scores are also elevated, except for β-strand 8.

Thus, our analysis suggests that the pLDDT scores can be closely linked to local protein dynamics, whereas the PAE scores capture large-scale conformational flexibility, with both scores being closely related to each other. We also find that relatively small errors in the pLDDT scores easily propagate large mismatches between the PAE matrix and global protein dynamics.

### Using AlphaFold Scores to Construct Elastic Network Models

To utilize the AlphaFold scores in explicit molecular dynamics simulations, we propose a framework to convert the statistical pLDDT and PAE scores into an elastic network model (ENM).^23−25^ An ENM can be constructed by adding harmonic potentials between, e.g., C_α_ atoms of the protein backbone of residues *i* and *j* according to
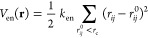
5where *V*_en_ is the total potential energy of the elastic network (en), *r*_c_ is the cutoff radius, and *k*_en_ is the force constant for the harmonic bond. The ENM additionally relies on reference distances *r*_*ij*_^0^ inferred from an initial reference structure, although this approach becomes inaccurate for proteins with multiple prominent conformational states. In the simplest formulation, a generic constant is used for all *k*_en_s. ENMs can also be generated based on short-timescale aMD simulations (in the order of tens of ns) in which both the number as well as the strength of bonds are optimized.^49−51^ However, many processes, such as the dissociation events and large-scale conformational changes, lie outside the short sampling timescales, making this approach less well suited for highly dynamical systems.^49^ Here, we propose an alternative strategy to adapt bonds and force constant based on AlphaFold scores.

For our AlphaFold-optimized elastic network model (AF-ENM), we suggest scaling the force constants based on the PAE scores. To this end, we assume that the distance distribution between the C_α_ atoms corresponds to a normal distribution, with the predicted aligned error as the standard deviation. The distance distribution *P* for a given distance between residues *i* and *j* can therefore be defined as
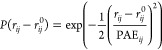
6Since the PAE matrix is generally not symmetrical, we use the average value between residue PAE*_ij_* and PAE*_ji_*. The relative free energy Δ*G*(*r*) can be calculated from the distance distribution
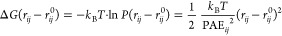
7By combining eqs 6 and 7, the force constant *k*_en_ for the bond between residues *i* and *j* can be defined as
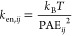
8where *k*_B_ is the Boltzmann constant and *T* is the temperature.

**Figure 3:**
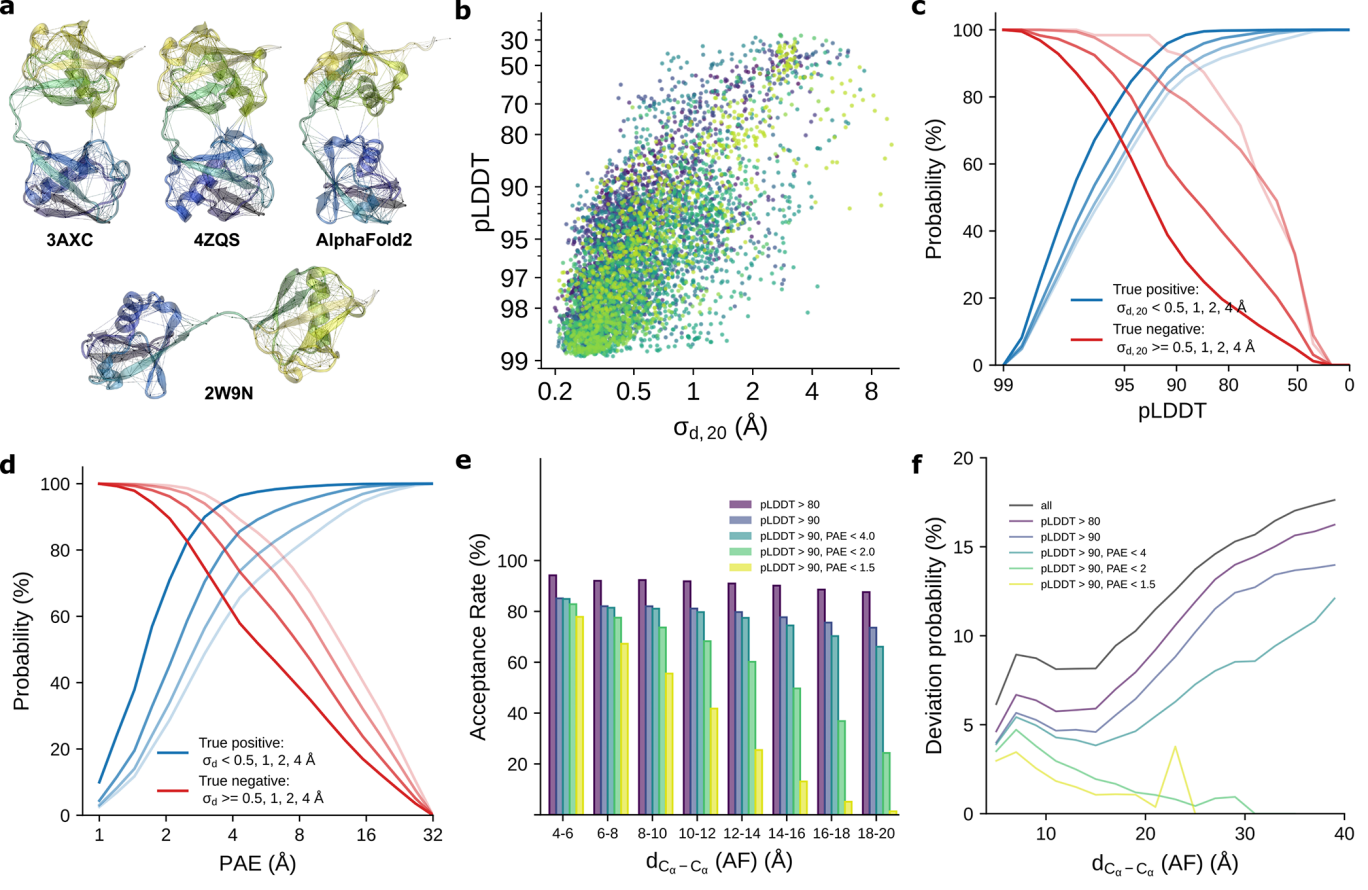
Construction of an elastic network model (ENM) based on AlphaFold for (a) diubiquitin (Ub_2_) ENMs with different starting structures. The protein is colored according to the residue number (from blue to yellow). The bonds and ENM (cutoff distance of 0.9 nm) between all C_α_ atoms are shown. (b) σ_d,20_ vs pLDDT scatter plots combined across all systems shown in Figure 1. (c) Sensitivity and specificity for the pLDDT/σ_d,20_ relations. The blue curves show the probability that a σ_d,20_ value is below a cutoff value (represented by different transparency levels) when the corresponding pLDDT score is above a threshold value (*x*-axis). The red curves show the probability that σ_d,20_ is *above* a cutoff value for residues with a pLDDT score below a threshold value. (d) Sensitivity and specificity for the σ_d_/PAE relationship. The blue curves show the probability that a PAE score (*x*-axis) is *below* a threshold value and is associated with a σ_d_ below a threshold value (represented by different transparency levels). The red curves show the probability that a PAE and associated σ_d_ are above threshold values (true negative test). (e) Probability that an AF predicted distance deviates by more than 20% from the average distance estimated by aMD under different selection criteria. (f) Ratio of aMD-derived distances that differ by more than 20% from the AlphaFold predicted structure.

ENMs are typically derived based on a single structure, but this can result in a significant underestimation of the global conformational dynamics. If multiple structures are available, it often remains unclear which of them (if any) would be suited to construct the ENM. For example, linear diubiquitin (Ub_2_, Figure S27) has different potential initial structures (Figure 3a). Ub_2_ is a highly flexible two-domain protein,^38,52^ in which the C-terminus of a proximal ubiquitin is linked to the N-terminus of a distal ubiquitin subunit. Different experimental structures have resolved Ub_2_ in both compact (PDB ID: 3AXC(53) and 4ZQS(52)) and open (PDB ID: 2W9N(54)) states, with Figure 3a also showing Ub_2_ as predicted by AlphaFold2.^1^ Depending on the initial conformations, the ENMs between the individual Ub-domains are vastly different, with the compact state (PDB ID: 4ZQS) having multiple bonds between the subunits, whereas the open model (PDB ID: 2W9N) shows no contacts.

To account for this bias, we propose to use the AlphaFold scores to remove network bonds with high expected fluctuation (Figure 3). To this end, we used the pLDDT scores to identify residues of the rigid protein cores (low σ_d,20_) and flexible/disordered regions (high σ_d,20_) that we further used to construct an optimized AF-ENM. We identify a threshold that balances the probability of finding a pLDDT score above a specific value, corresponding to a low σ_d,20_ (sensitivity or true positive rate) with the likelihood that the pLDDT score is below the threshold associated with a high σ_d,20_ value (specificity or true negative rate). The specificity and sensitivity show an inverse relationship (Figure 3c). We observe an excellent balance between them for a pLDDT threshold of 90, demonstrating a true positive rate of 86.5% and a true negative rate of 80.1% at a σ_d,20_ threshold of 2 Å as compared to 63.7 and 92.2% for a pLDDT threshold of 95, and 95.2 and 66.6% for a pLDDT threshold of 80.

While the pLDDT scores correlate with the local dynamics, the PAE scores are also good indicators of the global dynamics. We therefore use the PAEs to avoid over-constraining the conformational dynamics between two rigid regions in our AF-ENM. Similar to the dependency between pLDDT scores and σ_*d*,20_, we obtain a good balance between the sensitivity and specificity of the PAE/σ_d_ relationship (Figure 3d), as indicated by the likelihood that a low PAE score corresponds to a low σ_*d*_ (sensitivity or true positive test) relative to the probability that a high PAE value corresponds to a high σ_d_ (specificity or true negative test). As for the pLDDT σ_d,20_ relationship, we observe that the true positive and negative rates have an inverse relationship. Since the addition of a bond that spuriously restricts the dynamics is far more problematic than the absence of bonds in a rigid structure, we suggest that high sensitivity is more important than high specificity within the AF-ENM model. In this regard, we find that a PAE threshold of 2 Å provides a specificity of 98.8% and a sensitivity of 30.8%, compared to 99.8 and 14.0% for a PAE threshold of 1.5 Å, and 86.6 and 70.1% for a PAE threshold of 4 Å.

Finally, we also probed the effect of different *r*_*c*_ on our AF-ENMs. We find that the C_α_–C_α_ distances correlate with PAE values (Figure S30), with the likelihood of including a given bond in the AF-ENM strongly depending on the C_α_–C_α_ distances (Figure 3e). By only using a pLDDT threshold of 90, the acceptance rate is around 82% for C_α_–C_α_ distances between 8 and 10 Å and 73% for C_α_–C_α_ distances between 18 and 20 Å (Figure 3e). In contrast, when a PAE threshold of 2.0 Å is added, these values decrease to 74 and 24%, while a PAE threshold of 1.5 Å leads to 56 and 1.4%. To assess the extent to which the accepted bonds are representative of the structural ensemble, we examined the probability of the discrepancy of C_α_–C_α_ distances from AlphaFold and aMD simulations by more than 20% (Figure 3f). We observe that the deviation probability for a cutoff radius of 10 Å changes from 8.1 to 3.2% by including only distances with a pLDDT >90 and PAE <2 Å.

Based on these results, we used the following framework to construct AF-ENMs for MARTINI3 cgMD simulations: starting from an initial structure, we only considered residues with pLDDT scores >90 that are separated by at least two residues since the angle and bonded interactions account for their dynamics. Moreover, to balance sensitivity and specificity, we used a cutoff radius of 9 Å, with force constants determined by the PAE scores based on eq 8. For the AF-ENM cgMD simulations, we only consider bonds with *k* > 75 kJ/(mol·nm^2^), corresponding to a PAE threshold of 1.85 Å.

### Testing the AF-ENM Together with Martini3

To test the AF-ENM approach, we compared MARTINI3 cgMD simulations with a standard ENM using a generic *k*_en_ (500 kJ/(mol·nm^2^)), which is commonly used in MARTINI simulations,^23,55^ and the AF-ENM with a generic *k*_en_ and PAE-scaled *k*_en_ for three single-domain (protein 24–26, ubiquitin, lysozyme C, green fluorescent protein) and two proteins with two domains (protein 27 and 28, linear diubiquitin, NTD-MD construct of Hsp90) against aMD simulations. To estimate the difference in global dynamics, we performed principal component analysis (PCA) on the aMD trajectories (C_α_ only) to extract the two components with the highest variance.^56,57^ These components represent the proteins’ modes of motion with the most extensive conformational changes. We then project the backbone beads of the cgMD trajectories onto these modes and compare the resulting distributions with the multivariate Kullback–Leibler (KL) divergence^58,59^ (Figures S31–S35 and Table S4). A lower KL divergence corresponds to a higher similarity of two distributions and, therefore, a higher agreement between the global dynamics described by aMD and cgMD simulations.

Overall, we find that using AF-ENM instead of standard ENM leads to lower KL divergence (Table S4), with fairly low divergence between generic *k*_en_ and PAE-scaled *k*_en_. The difference is particularly pronounced for systems with higher conformational dynamics. For small rigid ubiquitin (Figure S31), we observe a KL divergence of 1.1 for the cgMD simulation with ENM compared to 1.8 with AF-ENM. In the case of lysozyme C (Figure S32), which has more flexible loop regions, we observe a reduction in KL divergence from 4.5 to 1.4. We also compared the experimental B-factors against the RMSF obtained by MD simulations^60^ and found an overall small improvement using AF-ENM as compared to the generic ENM (Table S5). GFP with the flexible N-terminal helix (Figure S33) shows a KL divergence of 11.0 and 4.8 for ENM and AF-ENM, a similar reduction as for the larger and flexible NTD and MD domains (Figure S35), where we notice a decrease from 11.9 to 1.2 and from 9.0 to 4.8. This is also consistent if comparing two domains, where we observe a KL divergence of 9.4 and 5.9 for linear diubiquitin (Figure S35) and 10.5 and 4.4 for the combined NTD-MD construct (Figure S35). The improvement is primarily connected to the reduction in bonds used in the ENM, as we observe similar KL divergences if we double the force constants for our AF-ENM simulations or use a generic, shared *k*_en_ (500 kJ/(mol·nm^2^)) (Table S4).

**Figure 4:**
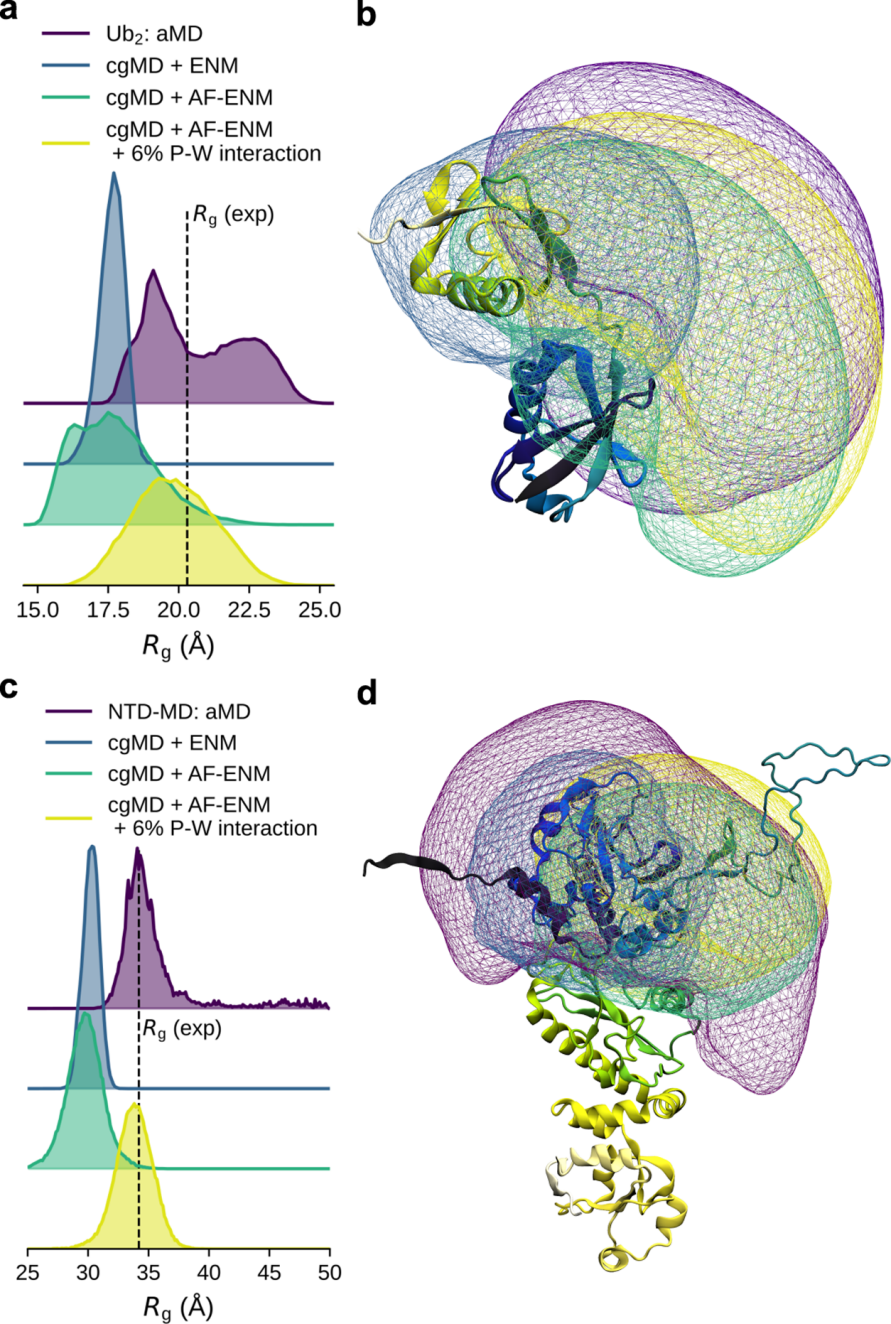
Comparison between aMD and cgMD simulations for linear Ub_2_ (protein 27) and the NTD-MD construct of Hsp90 (protein 28). (a, c) Radius of gyration (*R*_g_) distributions obtained by aMD and cgMD simulations for (a) linear Ub_2_ and (c) the Hsp90 construct. (b, d) Comparison of the conformational space occupied by (b) linear Ub_2_ and (d) the Hsp90 construct. The purple areas show the occupied space in the aMD simulations by the proximal ubiquitin relative to (b) the distal one and (d) the N-domain relative to the M domain of Hsp90. The blue areas show the occupied space in the cgMD simulations with ENF, the green areas correspond to the cgMD simulations with AF-ENM, while the yellow areas relate to the cgMD simulations with increased protein–water interactions.

The advantage of using the AF-ENM with MARTINI3 cgMD simulations is particularly evident when combined with increased protein–water (P–W) interactions.^35−39^ In Figure 4, we compare the radius of gyration (*R*_g_) distributions of our aMD ensembles with the cgMD simulations for linear Ub_2_ and the Hsp90 NTD-MD construct. Using a standard elastic network leads to an overly constrained and compact conformation, which fails to reproduce the experimental *R*_g_ value obtained from small-angle X-ray scattering (SAXS) experiments or the *R*_g_ distribution from aMD simulations. For Ub_2_, we observe an average *R*_g_ of 17.6 Å in our ENM simulation compared to 20.3 Å experimentally.^38^ At the same time, for the Hsp90 construct, the corresponding values are 30.2 and 34.5 Å.^42^ Using our AF-ENM increases conformational freedom and leads to broader *R*_g_ distributions centered around similar values compared to the ENM simulations (17.7 Å for Ub_2_ and 29.7 Å for NTD-MD construct). However, the increase in P–W interaction in combination with the AF-ENM simulations resulted in higher ratios of open conformations, which are in better agreement with aMD ensembles and experimental data, with average *R*_g_ values of 19.9 and 33.8 Å for Ub_2_ and the Hsp90 construct, respectively. Overall, combining the AF-ENM framework can improve the quality of the protein discerption in cgMD simulations relative to standard ENM. We find that the removal of the elastic network bonds between flexible regions is the main cause of the improvement while adding a PAE-based scaling of *k*_en_ provides only a secondary, much smaller improvement.

## Discussion

While AlphaFold is primarily used to predict protein structures, it is becoming increasingly clear that it can also be a valuable tool for inferring protein dynamics, as shown here (cf. also refs (11, 14, 15, 18, 19)). Here, we performed a comprehensive comparison of 28 different systems to link AlphaFold confidence scores to metrics obtained by atomistic molecular dynamics simulations. While the timescales of the aMD simulations are well suited to investigate the local fluctuation in the studied proteins, our simulations are not long enough to capture the full conformational ensemble of these systems, including possible unfolding and high barrier crossing events associated with different conformational states. Nevertheless, considering these limitations, we find that the pLDDT scores correlate with the local residual dynamics, while the PAE scores also provide insight into large-scale conformational dynamics. Although these scores are suited to unambiguously identify highly flexible and rigid protein regions and inter-domain interactions, accurately describing conditionally folded states remains challenging. We observe an overall strong correlation between prediction scores and the MD data, although the *R*-value varies widely between the individual systems. A substantial part of the structural uncertainty of AlphaFold is thus unlikely to arise only from the protein dynamics, with the difference in sequence depth between proteins and sub-regions causing additional uncertainty in pLDDT and PAE scores.

**Figure 5:**
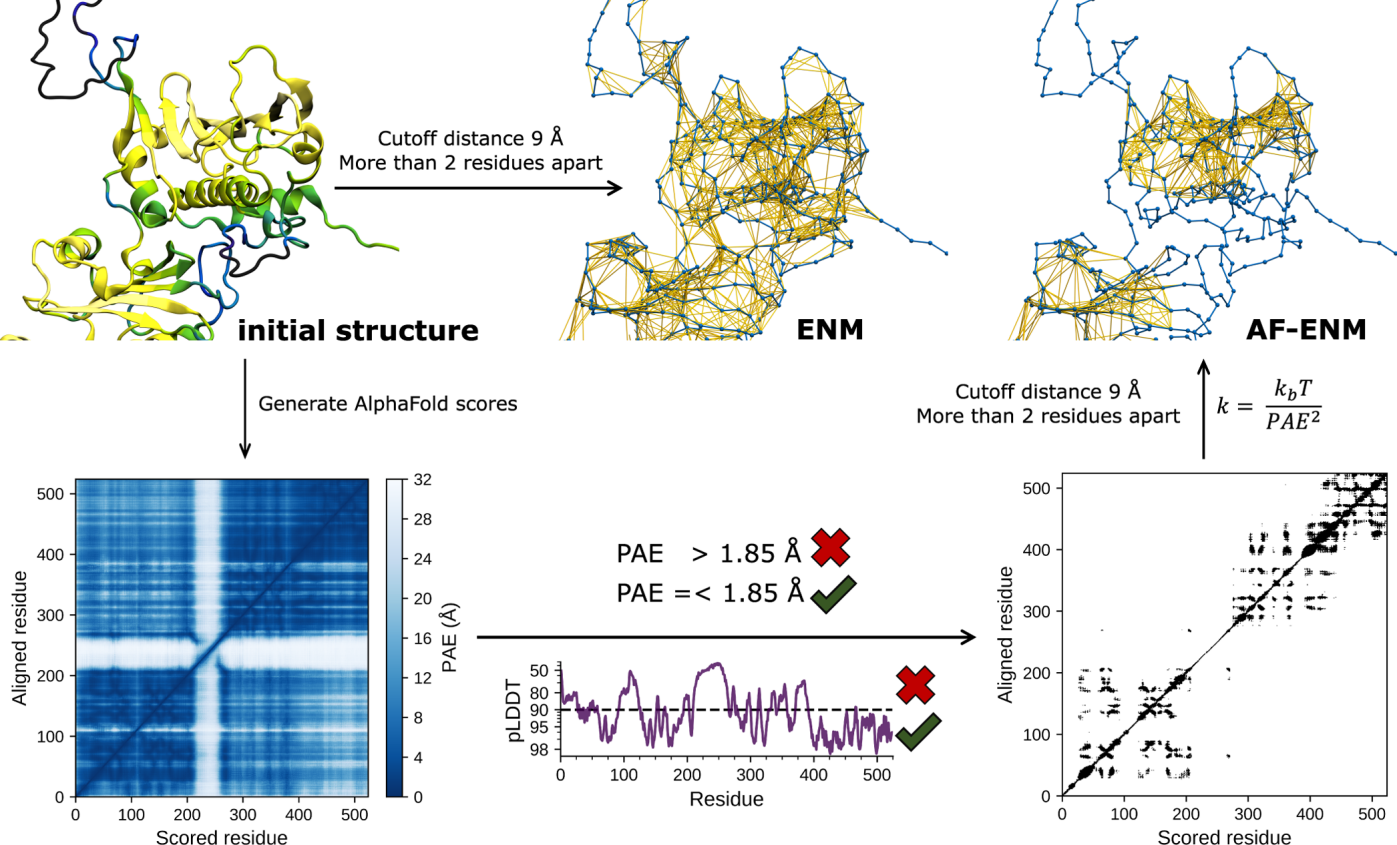
Schematic summary of the AF-ENM generation.

Our quantitative analysis allowed us to construct elastic networks based on these statistical scores (Figure 5) that can be effectively combined with cgMD simulations. For this, we take advantage of AlphaFold’s ability to identify flexible regions between rigid domains. We find that a pLDDT threshold of 90 is sufficient to reliably specify well-defined structural elements, which should be conserved by the network. A PAE threshold between 1.5 and 2.0 Å appears to be well suited to avoid overly restrictive bonds between rigid sections. Since the magnitude of the C_α_–C_α_ distance fluctuation correlates with the PAE score, the PAE matrix can be used to scale the force constants of the individual bonds. Our approach allows us to optimize an ENM with easily accessible or available data without relying on, e.g., additional atomistic simulations.^49−51^

In this study, we have tested the combination of the AF-ENM method with the MARTINI3 coarse-grained force field. The MARTINI model is widely used to study large-scale dynamics of complex systems due to its compatibility with a wide range of (bio-)molecules.^26,61,62^ However, the structure-based character and design of the MARTINI protein model may limit its application for the exploration of large-scale conformational changes in proteins, particularly if transitions in the secondary structure elements are involved. cgMD models and MARTINI3 are commonly employed together with an elastic network, which introduces a strong bias toward the starting conformation. Despite these limitations, the cgMD/MARTINI3 models are rather adaptable, with various approaches available to capture large-scale conformational changes of proteins and with applications even on highly complex intrinsically disordered proteins.^39,41^

Our AF-ENM model represents a significant improvement over the conventional elastic models commonly employed together with the MARTINI simulations, as the AlphaFold prediction scores allow us to identify flexible regions as well as dynamics between rigid domains. By implementing an elastic network only in the rigid domains/regions, we are able to preserve the structural integrity of tertiary and quaternary elements while minimizing restraint on large-scale protein dynamics. This approach may be particularly useful for studying complex membrane proteins, where the heterogeneous environment may impede the use of more accurate protein model or for the characterization of the extensive ensembles of flexible multidomain proteins. We also find that a further minor improvement in the MARTINI3-simulations can be achieved when the force constants are explicitly scaled based on the PAE matrix.

While we have here tested AF-ENM in combination with an ENM-dependent MARTIN3 coarse-grained force field,^29^ our approach could also increase the accuracy of other cgMD approaches, particularly of force fields that rely on sequence alignment data to create knowledge-based potentials (e.g., AWSEM-MD^63^). In this regard, homology and sequence information is used to determine native contacts or even energy functions.^64^ As the AlphaFold models are also based on sequence and homology data, the prediction scores might already encode this type of information. Furthermore, our AF-ENM method could be used with small modifications to analytically determine effective protein dynamics via normal mode analysis^65^ by primarily relying on the PAE scores to scale the force constants of the harmonic potentials. Although this approach would not be applicable to highly dynamic systems, it could nevertheless provide an improved description over conventional ENMs for rigid proteins. Similar to the AF-ENM framework, the AlphaFold scores could possibly be applied to derive Go̅-models,^40,41^ which have been used as an alternative for elastic networks in MARTINI simulations. Hereby, the harmonic potentials would be replaced by van der Waals interactions, whose strength and occurrence would be controlled based on pLDDT and PAE scores. This approach could overcome the observed limitations in conditionally folded structural elements, such as the β8-strand of Hsp90.^42^ The AF-ENM framework could thus open up new avenues to incorporate structural information into molecular dynamics simulations.

## Material and Methods

### MD Simulations

Atomistic molecular dynamics (aMD) simulations were performed for 28 proteins (Figure 1), as summarized in Table S1. The aMD simulations were performed with Gromacs^66^ using the a99SB-disp force field^43^ at *T* = 310 K, with a 2 fs timestep and with the protein models embedded in a water–ion environment with 100 mM NaCl. The temperature and pressure were controlled with the velocity rescaling thermostat^67^ and Parrinello–Rahman barostat.^68^ The two-domain proteins (1–23 and 27) were simulated for 2 μs, whereas the single-domain constructs (24–26) were simulated for 1 μs. For model 28, the NTD-MD construct of heat shock protein 90 (Hsp90), we used a 10.5 μs ensemble from our previous study,^42^ which was combined from 26 individual a99SB-disp simulations and reweighted with SAXS data based on a Bayesian/maximum entropy approach^69^ (see ref (42) for further details).

Coarse-grained MD (cgMD) simulations (Table S4) were created based on the atomistic models using the MARTINI3 coarse-grained force field.^29^ The cgMD models were embedded in a 100 mM NaCl solution. The simulations were performed at *T* = 310 K in an *NPT* ensemble with the velocity rescaling thermostat^67^ and Parrinello–Rahman barostat^68^ using Gromacs^66^ and 20 fs timesteps. The cgMD simulation was performed with different ENMs. As reference ENMs, we used a cutoff distance of 0.9 nm with 500 kJ/(mol·nm^2^). For our newly introduced AF-ENM approach, we used a shared *k*_en_ (500 kJ/(mol·nm^2^), AF-ENM (const)), *k*_en_s calculated with eq 8, and *k*_en_s with twice as large values as calculated with eq 8 (AF-ENMx2). Additional simulations were performed with AF-ENM and an extra 6% increased protein–water (P–W) interaction (AF-ENM + 6%). For the single-domain systems, cgMD simulations were performed with the standard ENM, AF-ENM, AF-ENM (const), and AF-ENMx2 for 10 μs each. For linear Ub_2_ and the NTD-MD construct, we ran 20 μs simulations with the standard ENM, AF-ENM, AF-ENM (const), AF-ENM + 6%, and AF-ENM + 6% (const).

## Supporting Information Available

Detailed comparison between the AlphaFold statistical scores and molecular dynamics simulations of the individual system; comparisons between aMD and cgMD simulations (PDF)

## Notes

The simulation trajectories can be found in Zenodo, 10.5281/zenodo.7212856.
