# Supplementary material for: Effective Molecular Dynamics from Neural Network-Based Structure Prediction Models
Source: J Chem Theory Comput. 2023 Mar 24;19(7):1965–75. doi: 10.1021/acs.jctc.2c01027 (PMC11181330; doi:10.1021/acs.jctc.2c01027)
Supplement: Supplementary file 1 — ct2c01027_si_001.pdf [file ct2c01027_si_001.pdf]

Supporting Information
Effective Molecular Dynamics from Neural-Network Based Structure Prediction Models

Alexander Jussupow[1], Ville R. I. Kaila[1,*]

[1] Department of Biochemistry and Biophysics, Stockholm University, 10691, Stockholm, Sweden.
*E-mail: ville.kaila@dbb.su.se

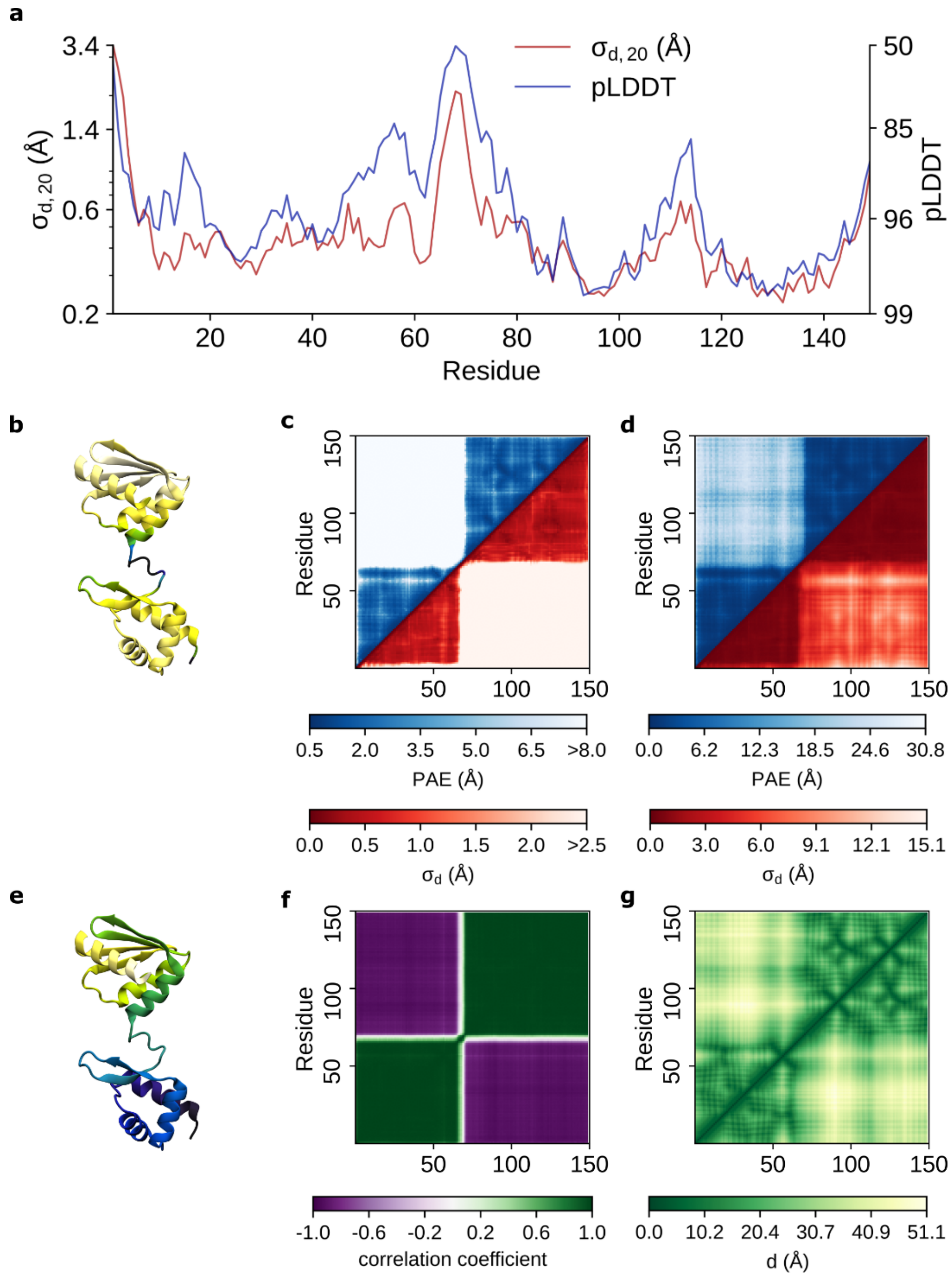

Figure S1: AlphaFold structure prediction vs. aMD of *arginine repressor* (protein 1) **a**) pLDDT scores vs. $\sigma_{d,20}$ values **b**) The protein structure colored based on its pLDDT scores. The dark blue colors correspond to residues with a pLDDT score <= 60, while the white color corresponds to residues with a pLDDT score close to 100. **c**) Comparison between (symmetrized) PAE matrices (blue) against the standard deviation of all C_α distances σ_d (red). The PAE scores range between 0.5 to 8.0 while the σ_d are limited to <2.5 Å. **d**) Comparison between (symmetrized) PAE matrices (blue) against the standard deviation of all C_α distances σ_d (red) for the maximum range. **e**) The protein structure colored based on its residue number. Dark blue colors correspond to low values, and yellows correspond to high values. **f**) Distance correlation matrix obtained from aMD simulations. **g**) Distance matrix obtained from aMD simulation.

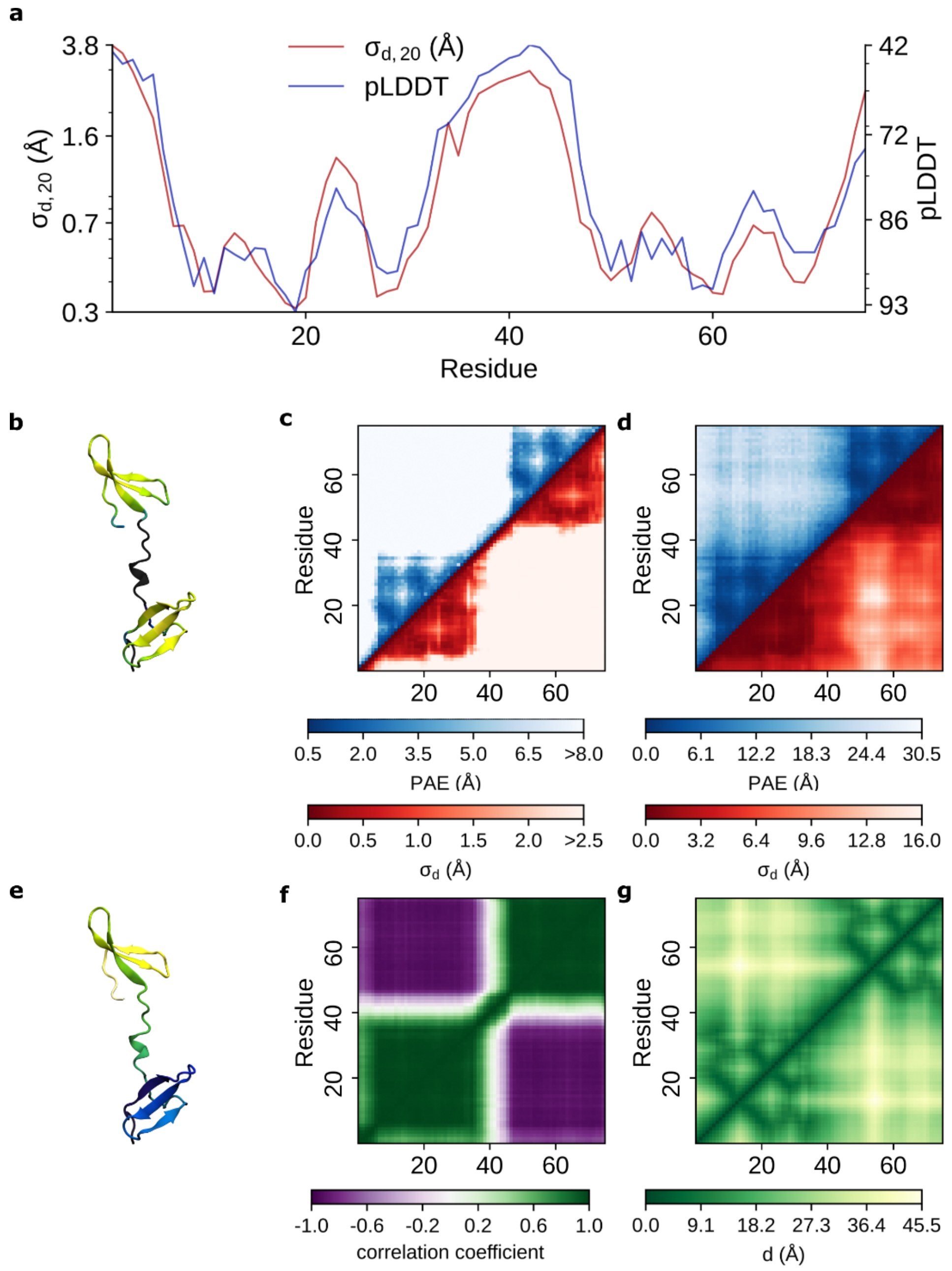

Figure S2: AlphaFold vs. aMD for *WW domain-binding protein 4* (protein 2) **a**) pLDDT scores vs. $\sigma_{d,20}$ values **b**) The protein structure colored based on its pLDDT scores. The dark blue colors correspond to residues with a pLDDT score <= 60, while the white color corresponds to residues with a pLDDT score close to 100. **c**) Comparison between (symmetrized) PAE matrices (blue) against the standard deviation of all C_α distances σ_d (red). The PAE scores range between 0.5 to 8.0 while the σ_d are limited to <2.5 Å. **d**) Comparison between (symmetrized) PAE matrices (blue) against the standard deviation of all C_α distances σ_d (red) for the maximum range. **e**) The protein structure colored based on its residue number. Dark blue colors correspond to low values, and yellows correspond to high values. **f**) Distance correlation matrix obtained from aMD simulations. **g**) Distance matrix obtained from aMD simulation.

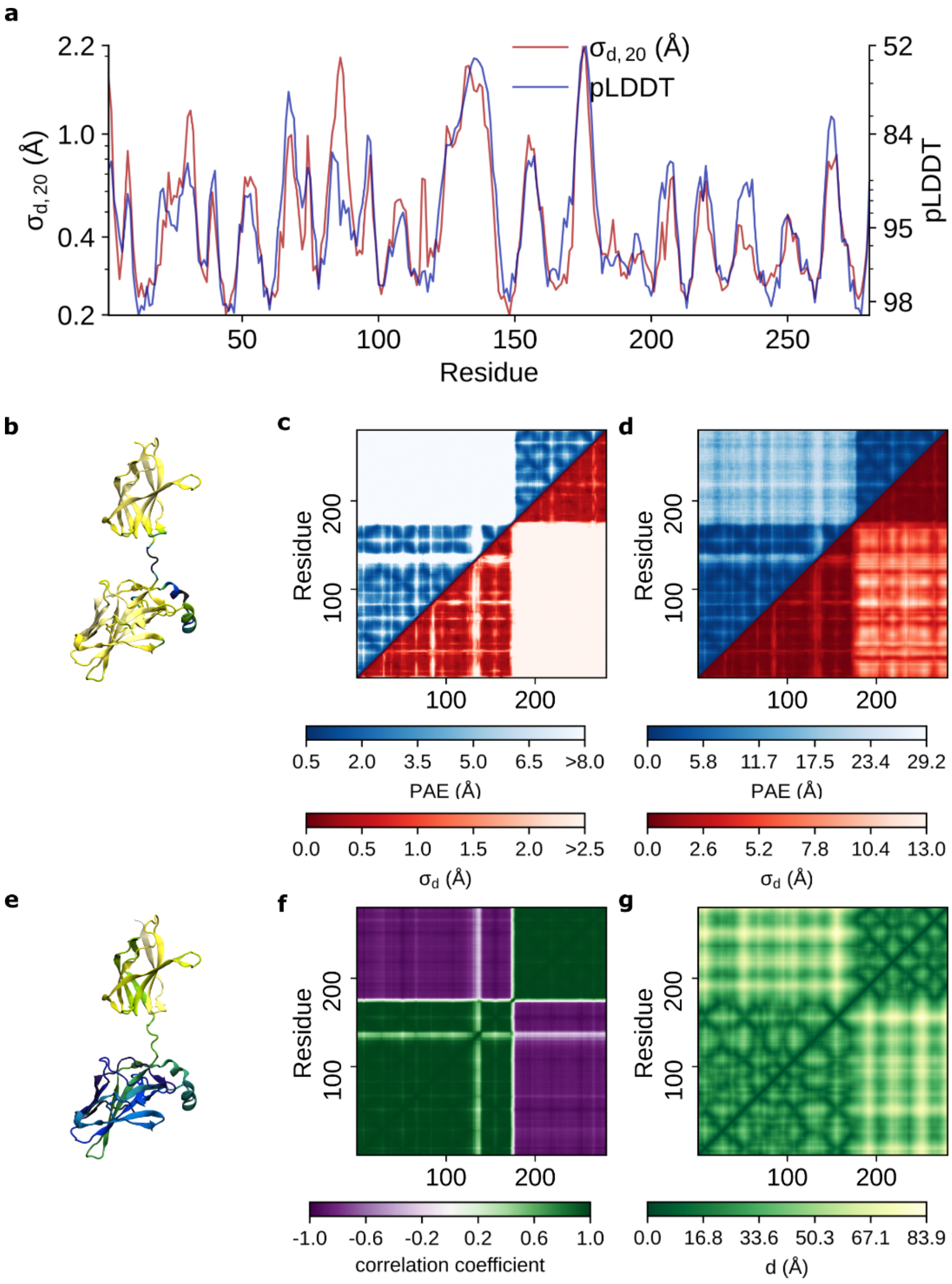

Figure S3: AlphaFold vs. aMD for *nuclear factor of activated T-cells, cytoplasmic 2* (protein 3) **a**) pLDDT scores vs. $\sigma_{d,20}$ values **b**) The protein structure colored based on its pLDDT scores. The dark blue colors correspond to residues with a pLDDT score <= 60, while the white color corresponds to residues with a pLDDT score close to 100. **c**) Comparison between (symmetrized) PAE matrices (blue) against the standard deviation of all C_α distances σ_d (red). The PAE scores range between 0.5 to 8.0 while the σ_d are limited to <2.5 Å. **d**) Comparison between (symmetrized) PAE matrices (blue) against the standard deviation of all C_α distances σ_d (red) for the maximum range. **e**) The protein structure colored based on its residue number. Dark blue colors correspond to low values, and yellows correspond to high values. **f**) Distance correlation matrix obtained from aMD simulations. **g**) Distance matrix obtained from aMD simulation.

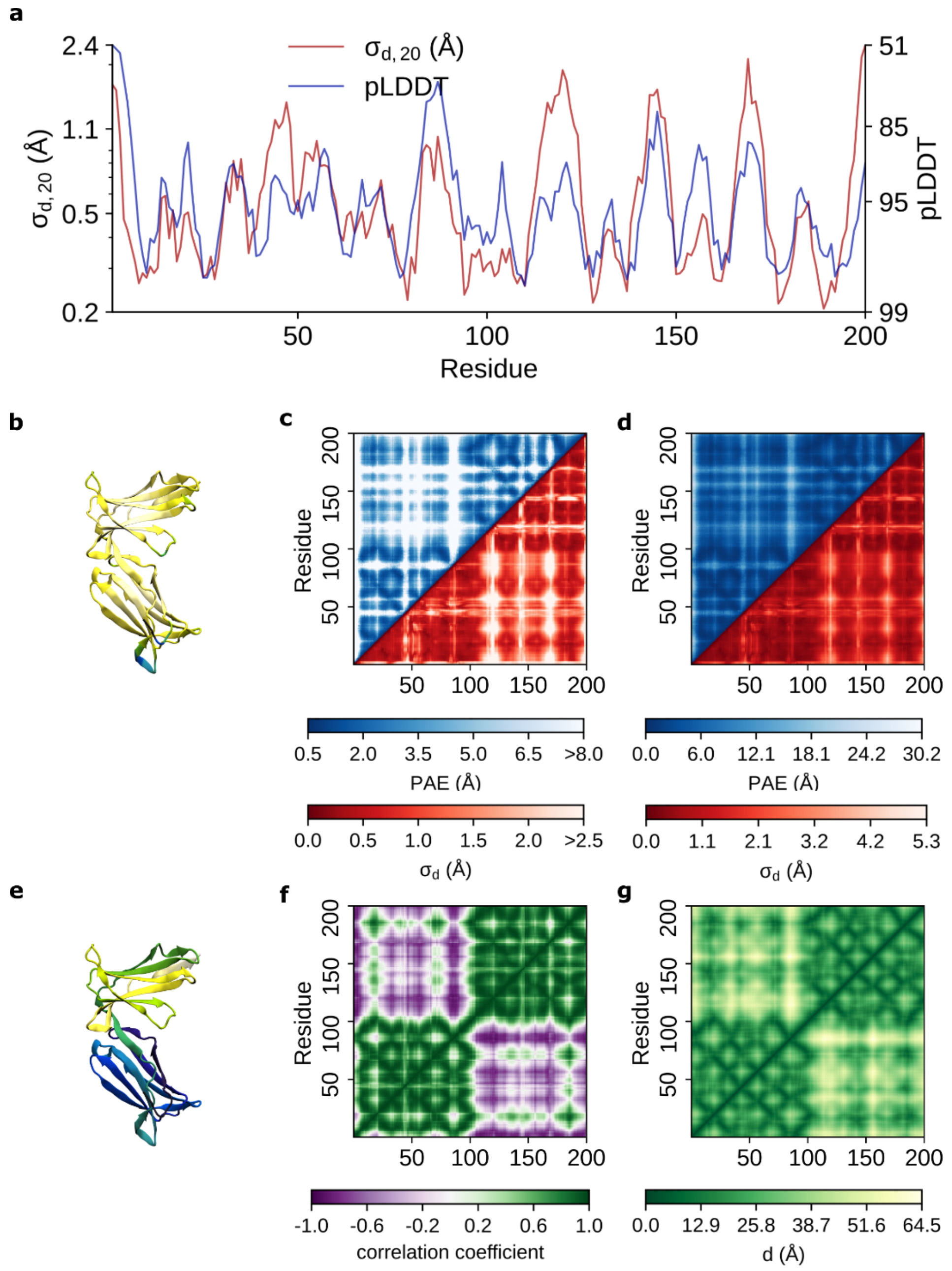

Figure S4: AlphaFold vs. aMD for *killer cell immunoglobulin-like receptor 2DL1* (protein 4) **a**) pLDDT scores vs. $\sigma_{d,20}$ values **b**) The protein structure colored based on its pLDDT scores. The dark blue colors correspond to residues with a pLDDT score <= 60, while the white color corresponds to residues with a pLDDT score close to 100. **c**) Comparison between (symmetrized) PAE matrices (blue) against the standard deviation of all C_α distances σ_d (red). The PAE scores range between 0.5 to 8.0 while the σ_d are limited to <2.5 Å. **d**) Comparison between (symmetrized) PAE matrices (blue) against the standard deviation of all C_α distances σ_d (red) for the maximum range. **e**) The protein structure colored based on its residue number. Dark blue colors correspond to low values, and yellows correspond to high values. **f**) Distance correlation matrix obtained from aMD simulations. **g**) Distance matrix obtained from aMD simulation.

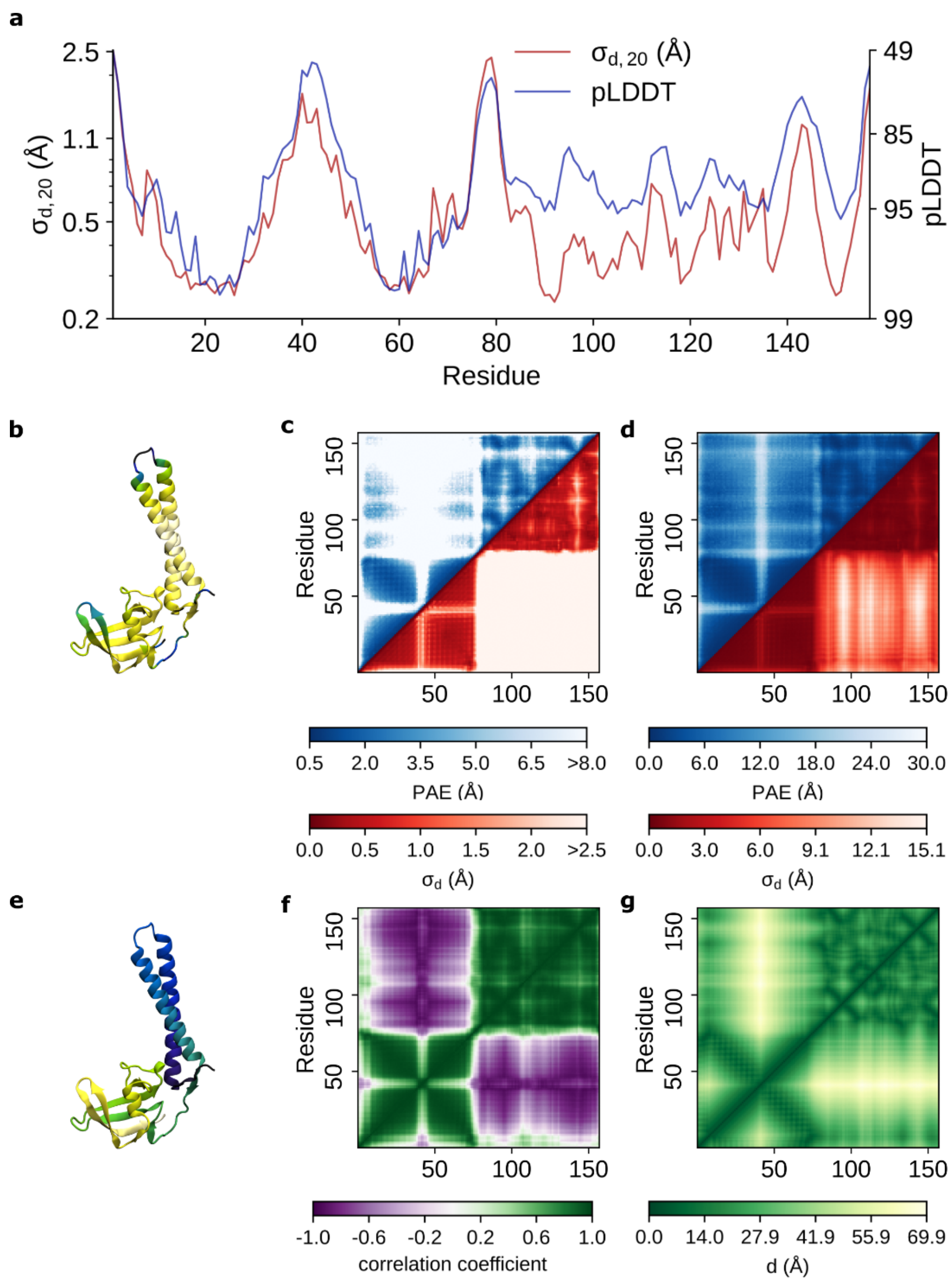

Figure S5: AlphaFold vs. aMD for *transcription inhibitor protein Gfh1* (protein 5) **a**) pLDDT scores vs. $\sigma_{d,20}$ values **b**) The protein structure colored based on its pLDDT scores. The dark blue colors correspond to residues with a pLDDT score <= 60, while the white color corresponds to residues with a pLDDT score close to 100. **c**) Comparison between (symmetrized) PAE matrices (blue) against the standard deviation of all C_α distances σ_d (red). The PAE scores range between 0.5 to 8.0 while the σ_d are limited to <2.5 Å. **d**) Comparison between (symmetrized) PAE matrices (blue) against the standard deviation of all C_α distances σ_d (red) for the maximum range. **e**) The protein structure colored based on its residue number. Dark blue colors correspond to low values, and yellows correspond to high values. **f**) Distance correlation matrix obtained from aMD simulations. **g**) Distance matrix obtained from aMD simulation.

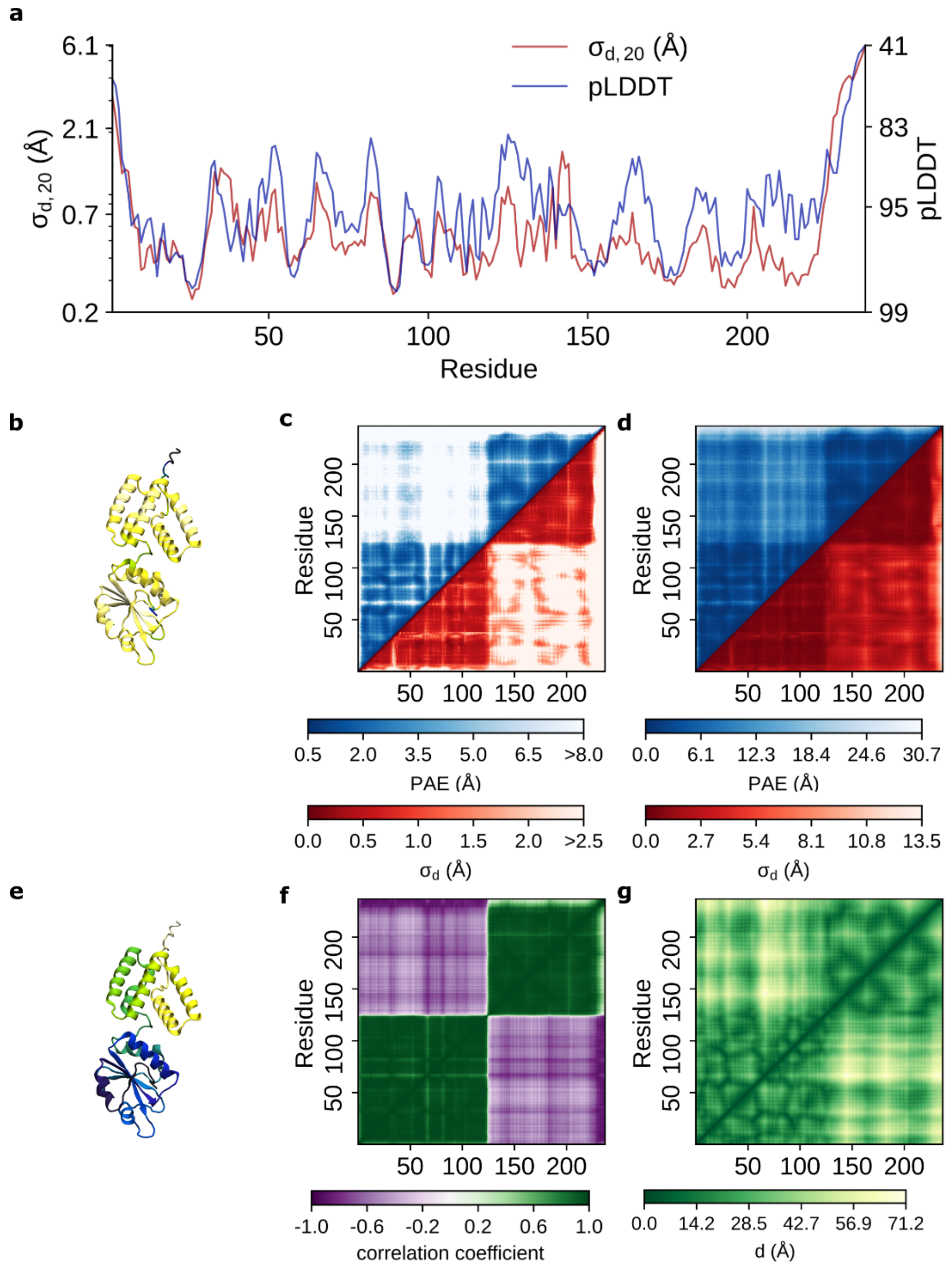

Figure S6: AlphaFold vs. aMD for *protein windbeutel* (protein 6) **a**) pLDDT scores vs. $\sigma_{d,20}$ values **b**) The protein structure colored based on its pLDDT scores. The dark blue colors correspond to residues with a pLDDT score <= 60, while the white color corresponds to residues with a pLDDT score close to 100. **c**) Comparison between (symmetrized) PAE matrices (blue) against the standard deviation of all C_α distances σ_d (red). The PAE scores range between 0.5 to 8.0 while the σ_d are limited to <2.5 Å. **d**) Comparison between (symmetrized) PAE matrices (blue) against the standard deviation of all C_α distances σ_d (red) for the maximum range. **e**) The protein structure colored based on its residue number. Dark blue colors correspond to low values, and yellows correspond to high values. **f**) Distance correlation matrix obtained from aMD simulations. **g**) Distance matrix obtained from aMD simulation.

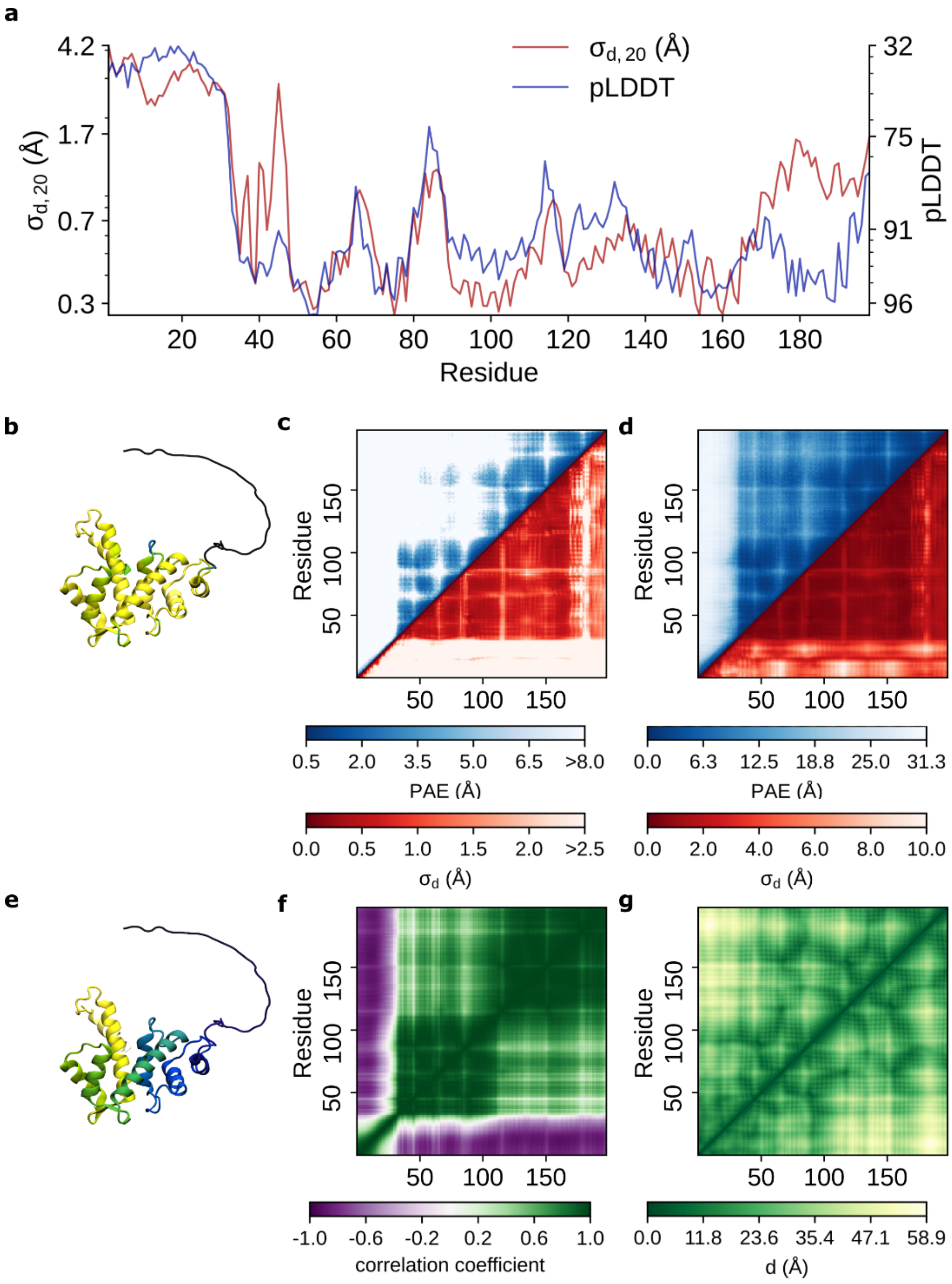

Figure S7: AlphaFold vs. *aMD for sorcin* (protein 7) **a**) pLDDT scores vs. $\sigma_{d,20}$ values **b**) The protein structure colored based on its pLDDT scores. The dark blue colors correspond to residues with a pLDDT score <= 60, while the white color corresponds to residues with a pLDDT score close to 100. **c**) Comparison between (symmetrized) PAE matrices (blue) against the standard deviation of all C_α distances σ_d (red). The PAE scores range between 0.5 to 8.0 while the σ_d are limited to <2.5 Å. **d**) Comparison between (symmetrized) PAE matrices (blue) against the standard deviation of all C_α distances σ_d (red) for the maximum range. **e**) The protein structure colored based on its residue number. Dark blue colors correspond to low values, and yellows correspond to high values. **f**) Distance correlation matrix obtained from aMD simulations. **g**) Distance matrix obtained from aMD simulation.

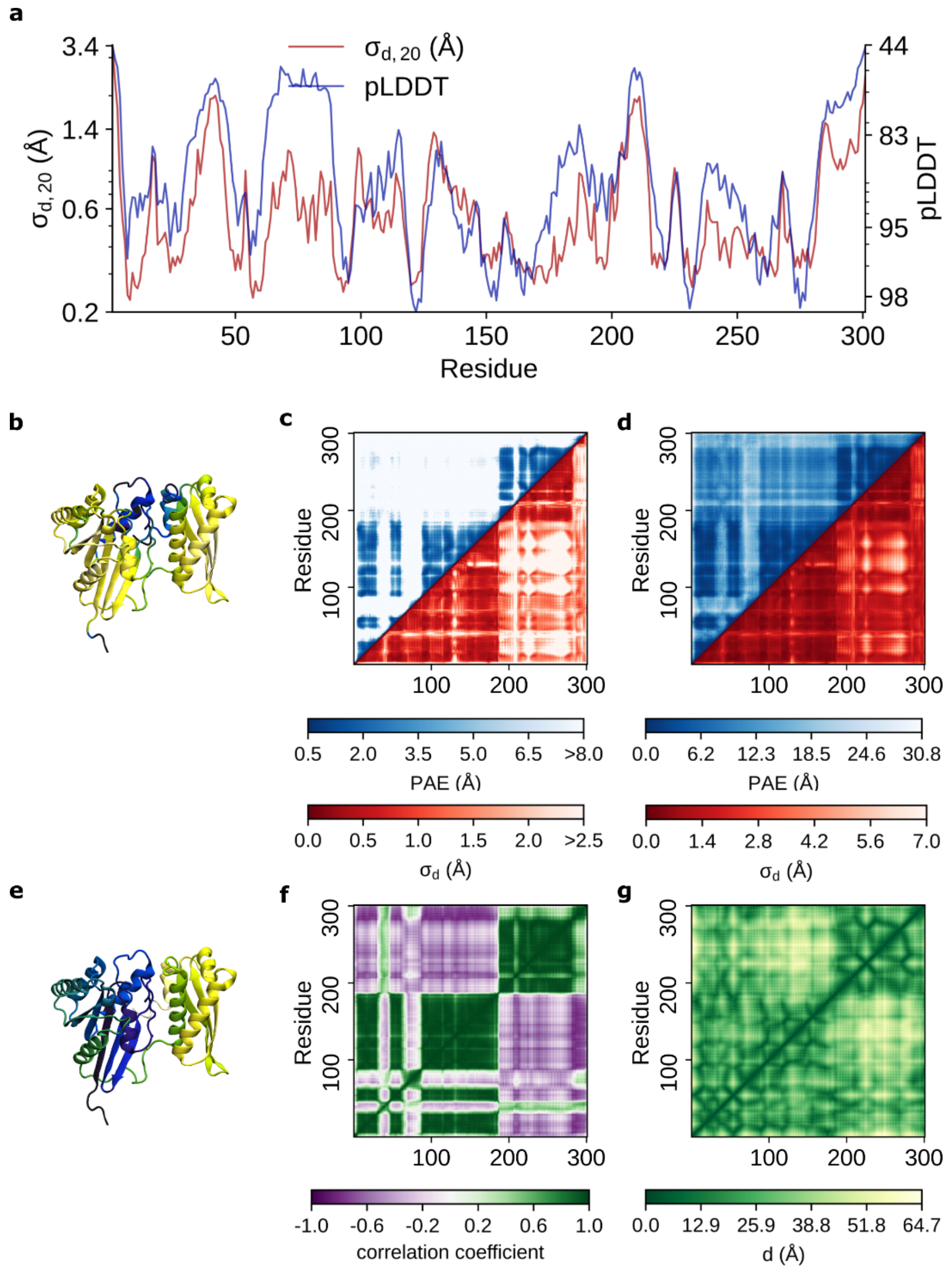

Figure S8: AlphaFold vs. aMD for *GTPase Era* (protein 8) **a**) pLDDT scores vs. $\sigma_{d,20}$ values **b**) The protein structure colored based on its pLDDT scores. The dark blue colors correspond to residues with a pLDDT score <= 60, while the white color corresponds to residues with a pLDDT score close to 100. **c**) Comparison between (symmetrized) PAE matrices (blue) against the standard deviation of all C_α distances σ_d (red). The PAE scores range between 0.5 to 8.0 while the σ_d are limited to <2.5 Å. **d**) Comparison between (symmetrized) PAE matrices (blue) against the standard deviation of all C_α distances σ_d (red) for the maximum range. **e**) The protein structure colored based on its residue number. Dark blue colors correspond to low values, and yellows correspond to high values. **f**) Distance correlation matrix obtained from aMD simulations. **g**) Distance matrix obtained from aMD simulation.

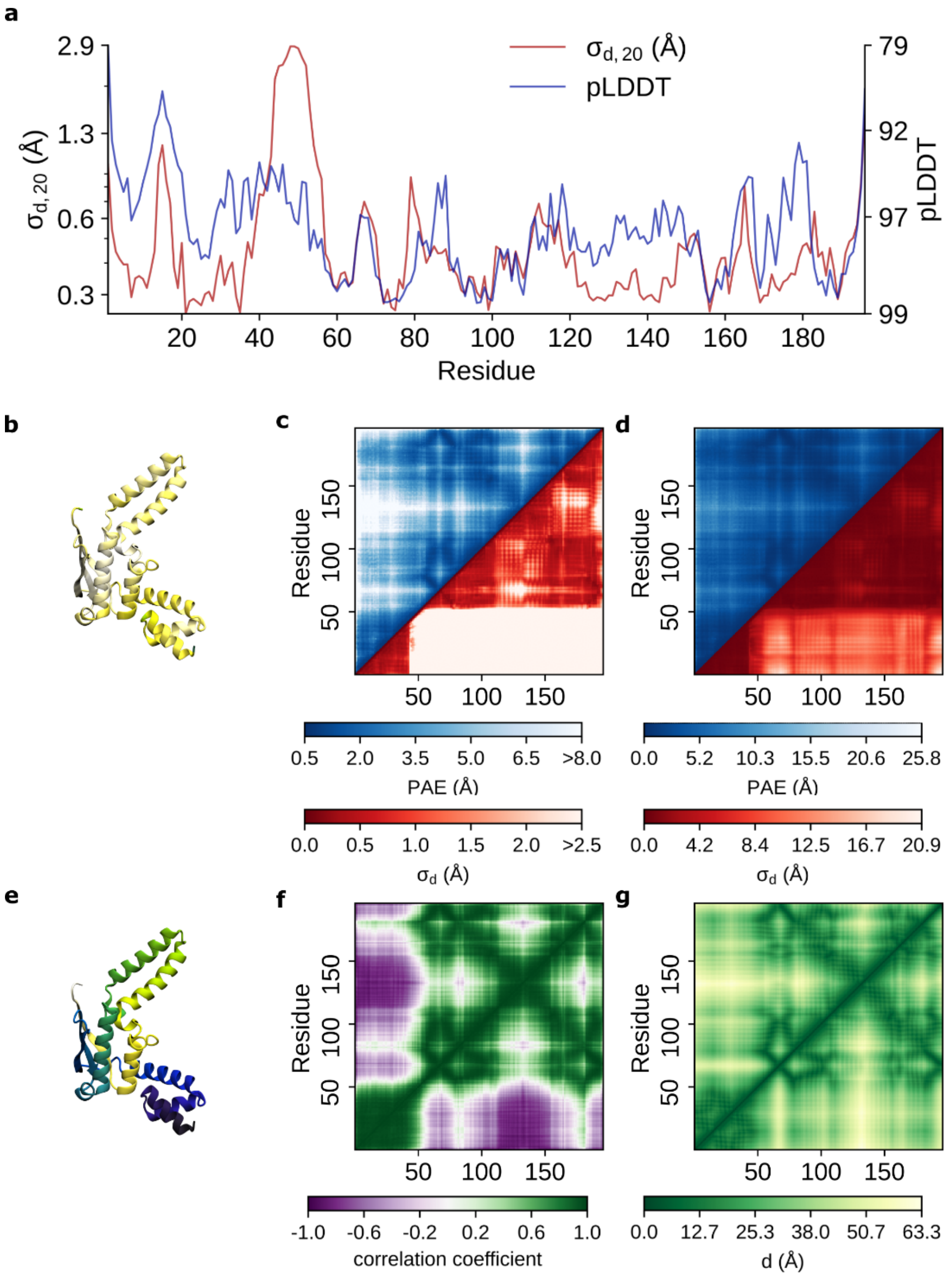

Figure S9: AlphaFold vs. aMD for *elongation factor T* (protein 9) **a**) pLDDT scores vs. $\sigma_{d,20}$ values **b**) The protein structure colored based on its pLDDT scores. The dark blue colors correspond to residues with a pLDDT score <= 60, while the white color corresponds to residues with a pLDDT score close to 100. **c**) Comparison between (symmetrized) PAE matrices (blue) against the standard deviation of all C_α distances σ_d (red). The PAE scores range between 0.5 to 8.0 while the σ_d are limited to <2.5 Å. **d**) Comparison between (symmetrized) PAE matrices (blue) against the standard deviation of all C_α distances σ_d (red) for the maximum range. **e**) The protein structure colored based on its residue number. Dark blue colors correspond to low values, and yellows correspond to high values. **f**) Distance correlation matrix obtained from aMD simulations. **g**) Distance matrix obtained from aMD simulation.

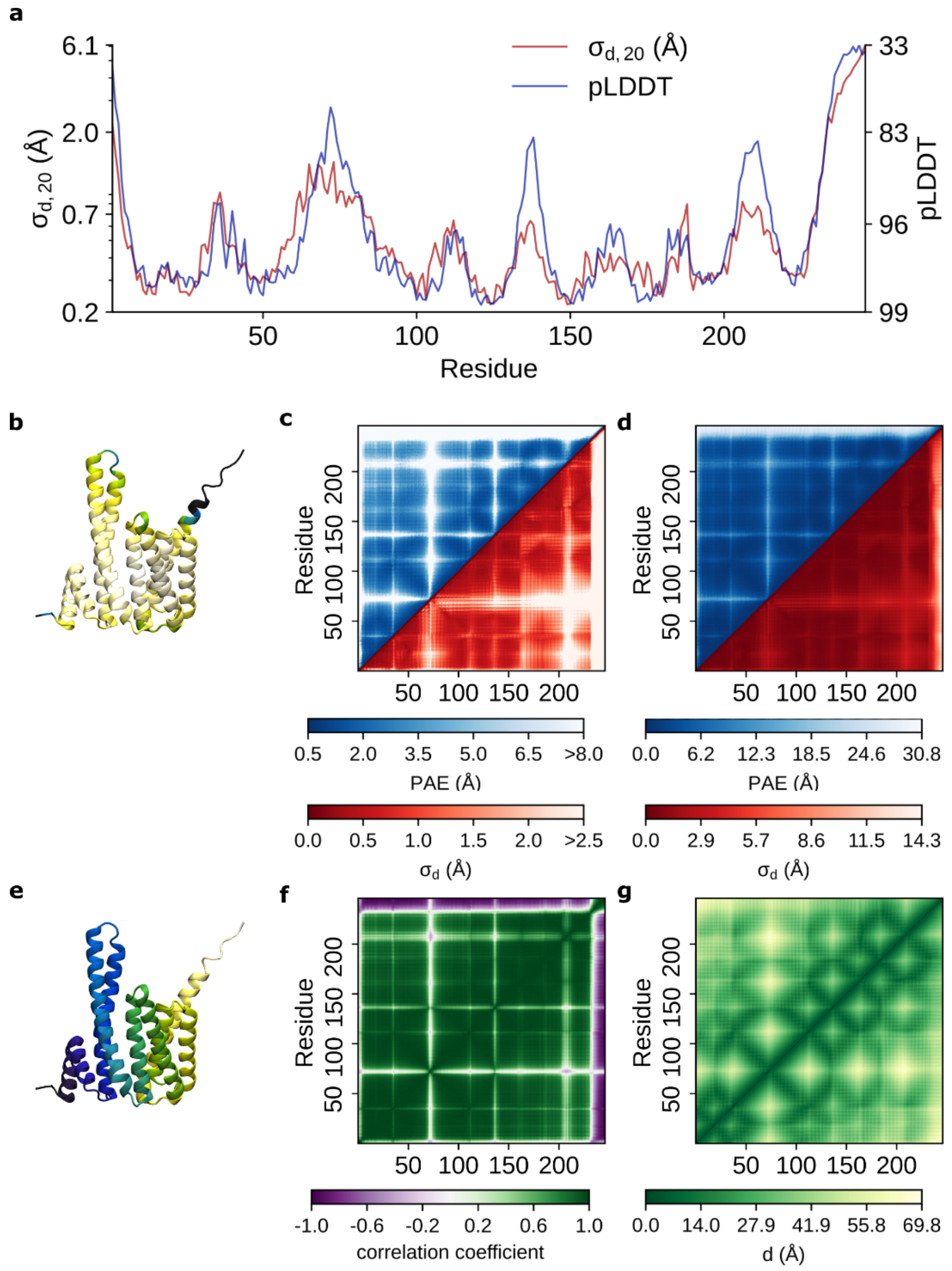

Figure S10: AlphaFold vs. aMD for *14-3-3 protein beta/alpha* (protein 10) **a**) pLDDT scores vs. $\sigma_{d,20}$ values **b**) The protein structure colored based on its pLDDT scores. The dark blue colors correspond to residues with a pLDDT score <= 60, while the white color corresponds to residues with a pLDDT score close to 100. **c**) Comparison between (symmetrized) PAE matrices (blue) against the standard deviation of all C_α distances σ_d (red). The PAE scores range between 0.5 to 8.0 while the σ_d are limited to <2.5 Å. **d**) Comparison between (symmetrized) PAE matrices (blue) against the standard deviation of all C_α distances σ_d (red) for the maximum range. **e**) The protein structure colored based on its residue number. Dark blue colors correspond to low values, and yellows correspond to high values. **f**) Distance correlation matrix obtained from aMD simulations. **g**) Distance matrix obtained from aMD simulation.

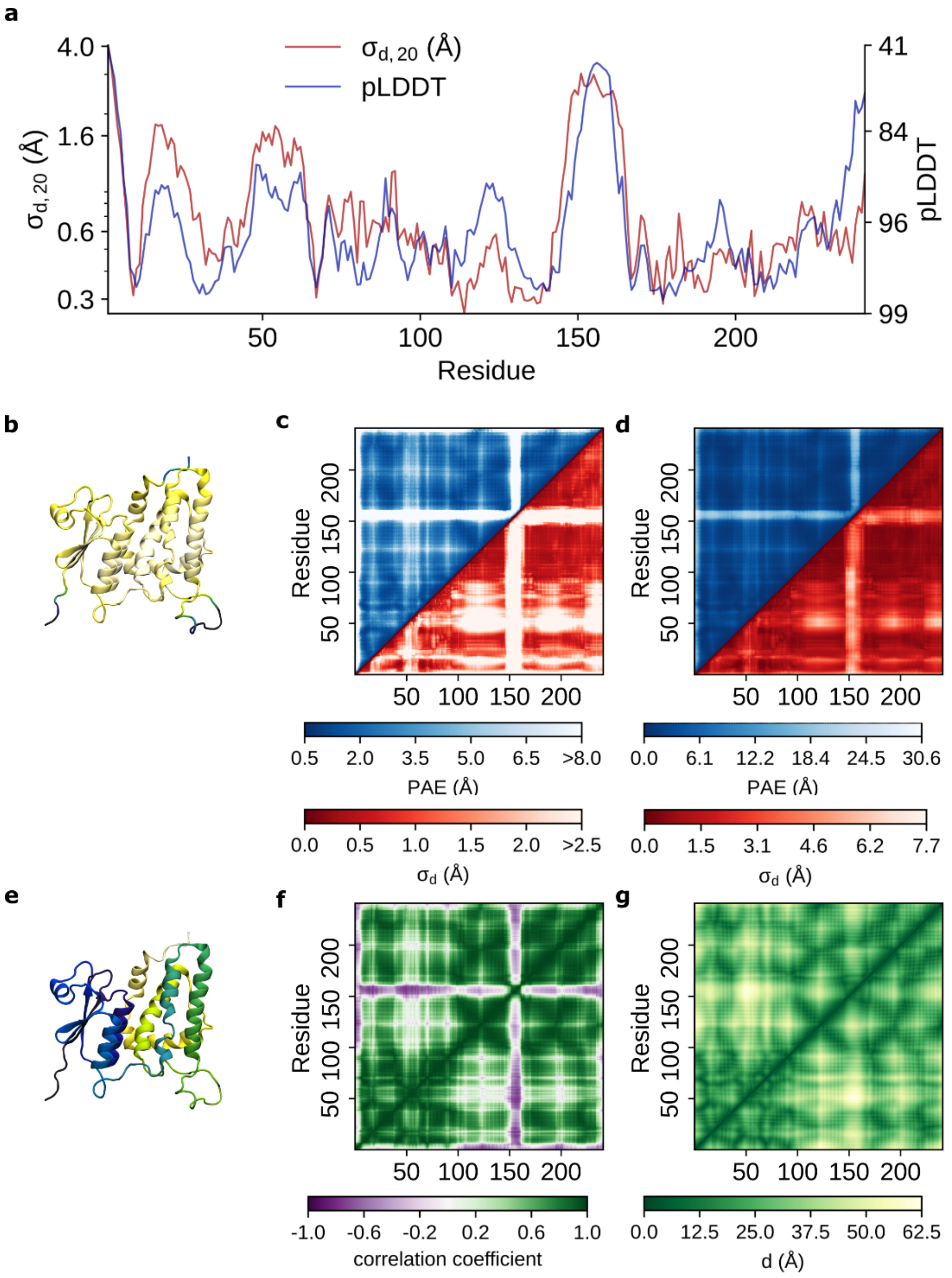

Figure S11: AlphaFold vs. aMD for *chloride intracellular channel protein 1* (protein 11) **a**) pLDDT scores vs. $\sigma_{d,20}$ values **b**) The protein structure colored based on its pLDDT scores. The dark blue colors correspond to residues with a pLDDT score <= 60, while the white color corresponds to residues with a pLDDT score close to 100. **c**) Comparison between (symmetrized) PAE matrices (blue) against the standard deviation of all C_α distances σ_d (red). The PAE scores range between 0.5 to 8.0 while the σ_d are limited to <2.5 Å. **d**) Comparison between (symmetrized) PAE matrices (blue) against the standard deviation of all C_α distances σ_d (red) for the maximum range. **e**) The protein structure colored based on its residue number. Dark blue colors correspond to low values, and yellows correspond to high values. **f**) Distance correlation matrix obtained from aMD simulations. **g**) Distance matrix obtained from aMD simulation.

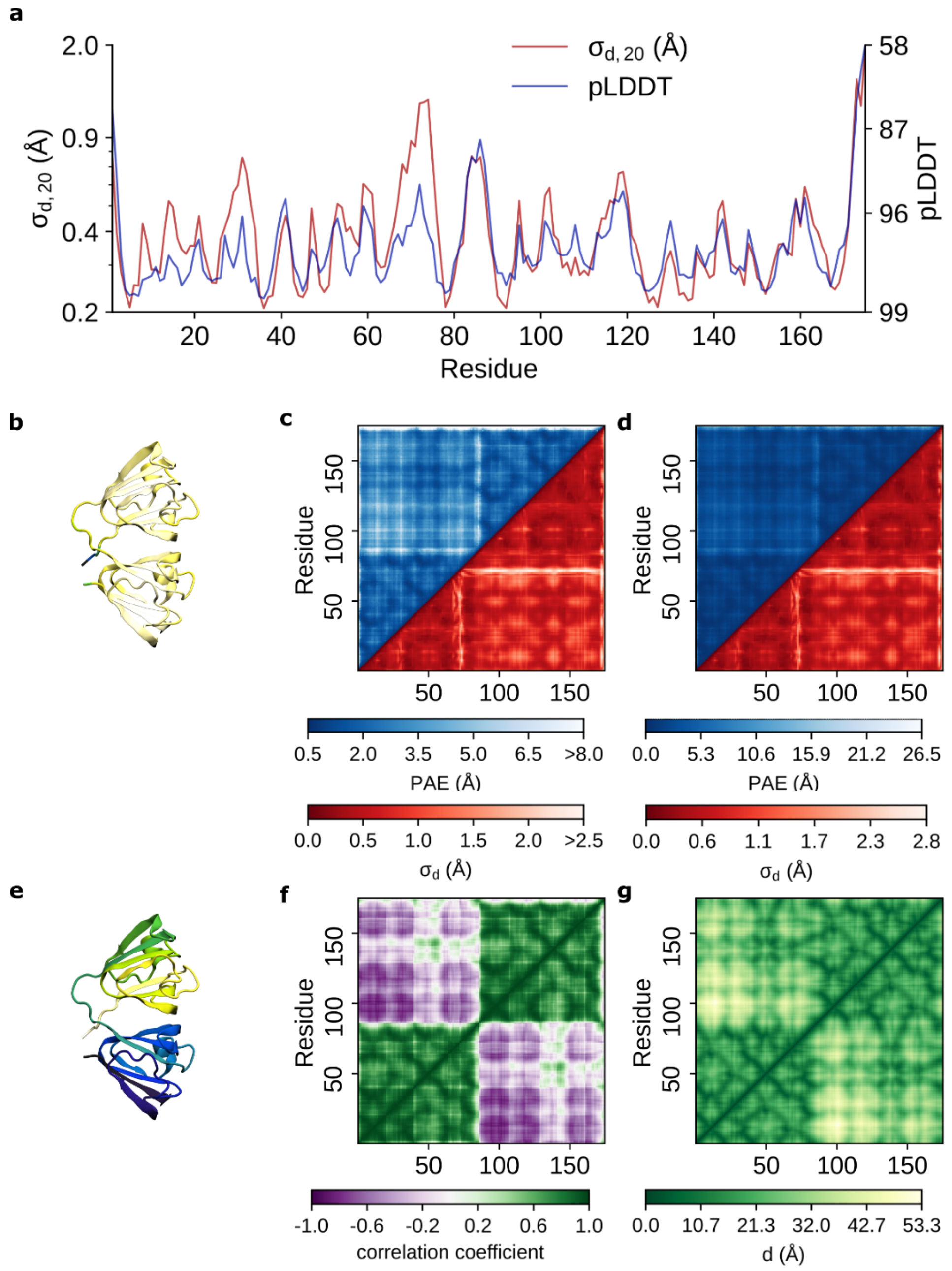

Figure S12: AlphaFold vs. aMD for *gamma-crystallin B* (protein 12) **a**) pLDDT scores vs. $\sigma_{d,20}$ values **b**) The protein structure colored based on its pLDDT scores. The dark blue colors correspond to residues with a pLDDT score <= 60, while the white color corresponds to residues with a pLDDT score close to 100. **c**) Comparison between (symmetrized) PAE matrices (blue) against the standard deviation of all C_α distances σ_d (red). The PAE scores range between 0.5 to 8.0 while the σ_d are limited to <2.5 Å. **d**) Comparison between (symmetrized) PAE matrices (blue) against the standard deviation of all C_α distances σ_d (red) for the maximum range. **e**) The protein structure colored based on its residue number. Dark blue colors correspond to low values, and yellows correspond to high values. **f**) Distance correlation matrix obtained from aMD simulations. **g**) Distance matrix obtained from aMD simulation.

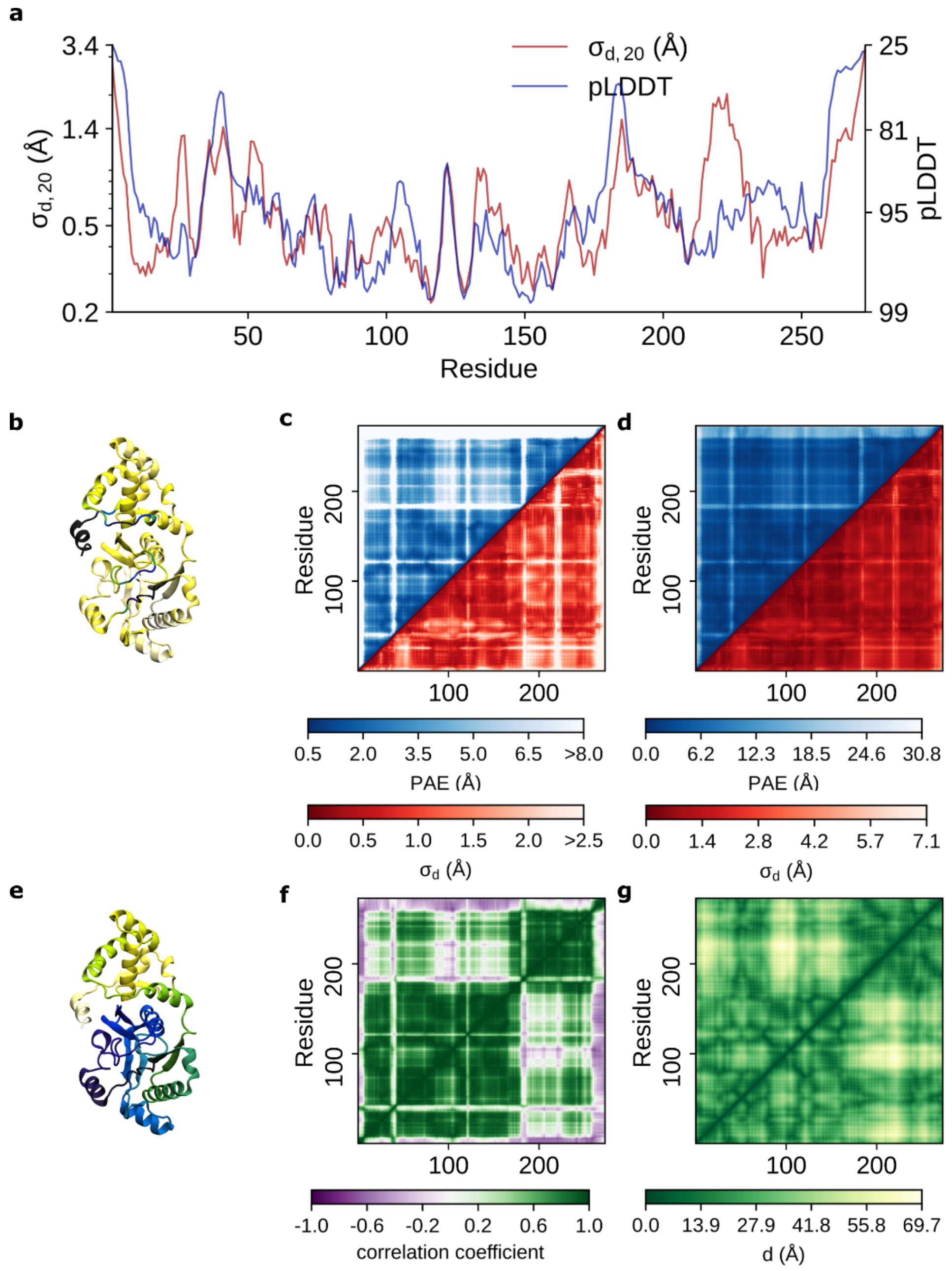

Figure S13: AlphaFold vs. aMD for *transposon Tn7 transposition protein TnsA* (protein 13) **a**) pLDDT scores vs. $\sigma_{d,20}$ values **b**) The protein structure colored based on its pLDDT scores. The dark blue colors correspond to residues with a pLDDT score <= 60, while the white color corresponds to residues with a pLDDT score close to 100. **c**) Comparison between (symmetrized) PAE matrices (blue) against the standard deviation of all C_α distances σ_d (red). The PAE scores range between 0.5 to 8.0 while the σ_d are limited to <2.5 Å. **d**) Comparison between (symmetrized) PAE matrices (blue) against the standard deviation of all C_α distances σ_d (red) for the maximum range. **e**) The protein structure colored based on its residue number. Dark blue colors correspond to low values, and yellows correspond to high values. **f**) Distance correlation matrix obtained from aMD simulations. **g**) Distance matrix obtained from aMD simulation.

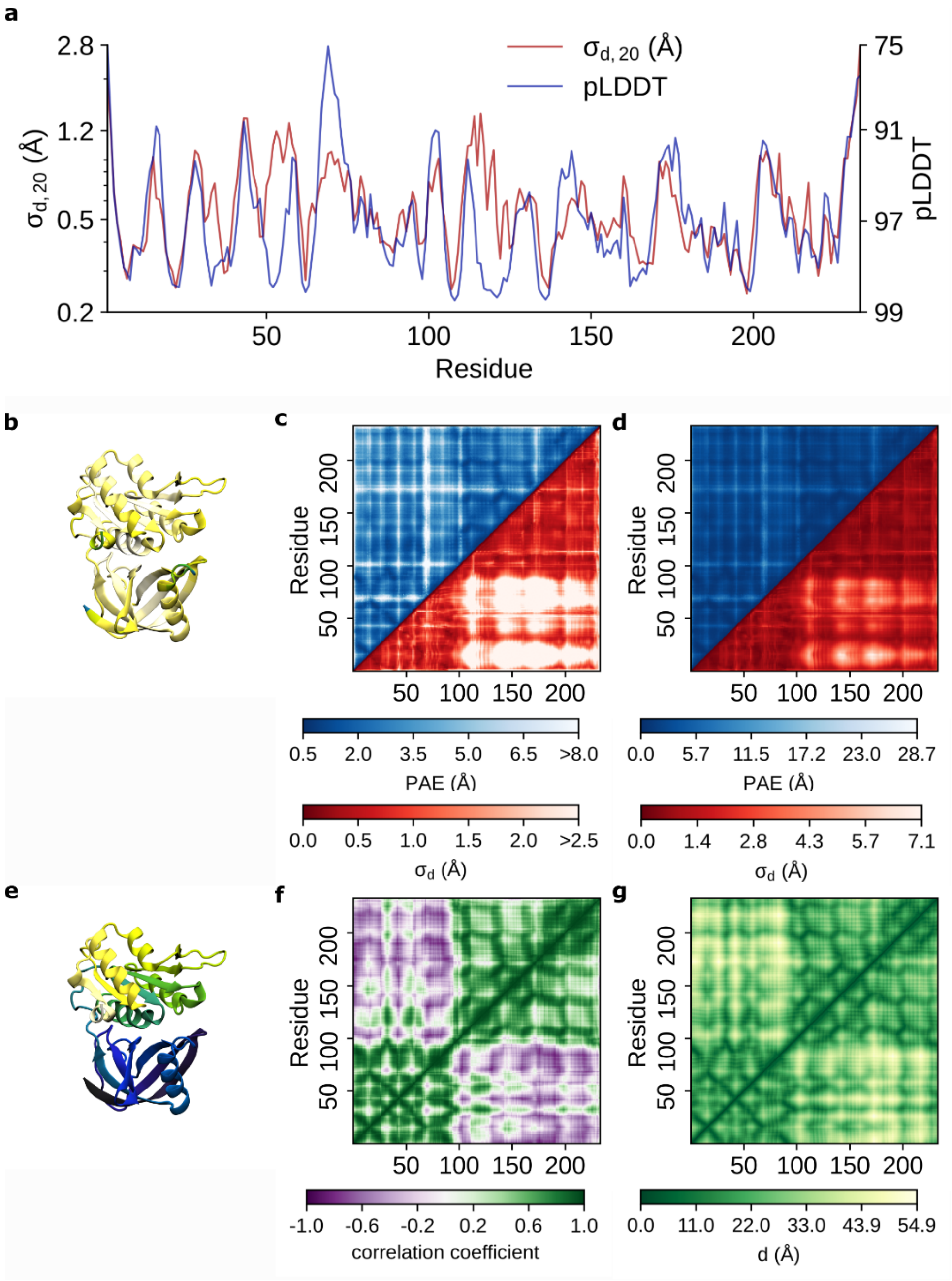

Figure S14: AlphaFold vs. aMD for *NAD(P)H-flavin reductase* (protein 14) **a**) pLDDT scores vs. $\sigma_{d,20}$ values **b**) The protein structure colored based on its pLDDT scores. The dark blue colors correspond to residues with a pLDDT score <= 60, while the white color corresponds to residues with a pLDDT score close to 100. **c**) Comparison between (symmetrized) PAE matrices (blue) against the standard deviation of all C_α distances σ_d (red). The PAE scores range between 0.5 to 8.0 while the σ_d are limited to <2.5 Å. **d**) Comparison between (symmetrized) PAE matrices (blue) against the standard deviation of all C_α distances σ_d (red) for the maximum range. **e**) The protein structure colored based on its residue number. Dark blue colors correspond to low values, and yellows correspond to high values. **f**) Distance correlation matrix obtained from aMD simulations. **g**) Distance matrix obtained from aMD simulation.

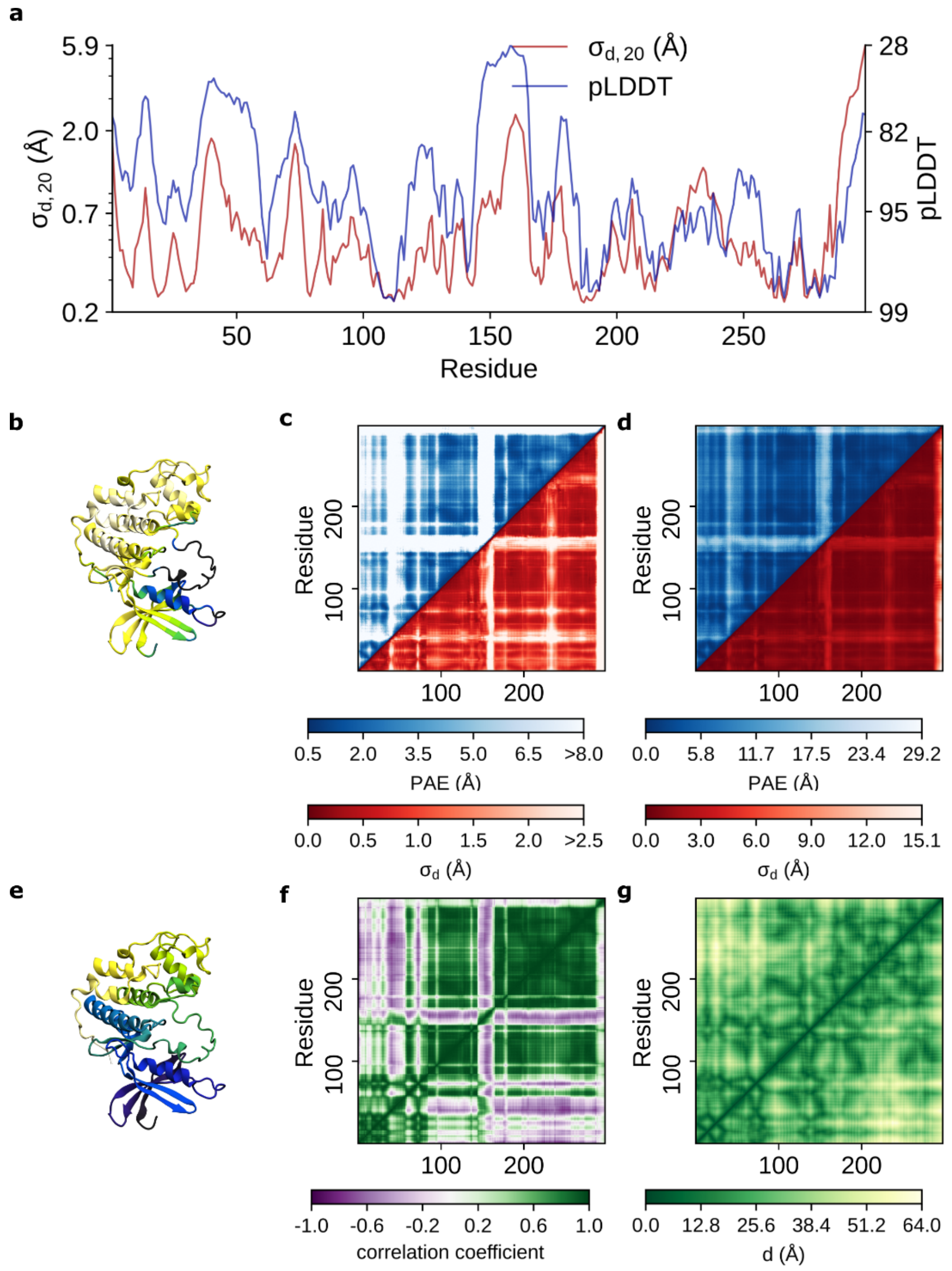

Figure S15: AlphaFold vs. aMD for *cyclin-dependent kinase 2* (protein 15) **a**) pLDDT scores vs. $\sigma_{d,20}$ values **b**) The protein structure colored based on its pLDDT scores. The dark blue colors correspond to residues with a pLDDT score <= 60, while the white color corresponds to residues with a pLDDT score close to 100. **c**) Comparison between (symmetrized) PAE matrices (blue) against the standard deviation of all C_α distances σ_d (red). The PAE scores range between 0.5 to 8.0 while the σ_d are limited to <2.5 Å. **d**) Comparison between (symmetrized) PAE matrices (blue) against the standard deviation of all C_α distances σ_d (red) for the maximum range. **e**) The protein structure colored based on its residue number. Dark blue colors correspond to low values, and yellows correspond to high values. **f**) Distance correlation matrix obtained from aMD simulations. **g**) Distance matrix obtained from aMD simulation.

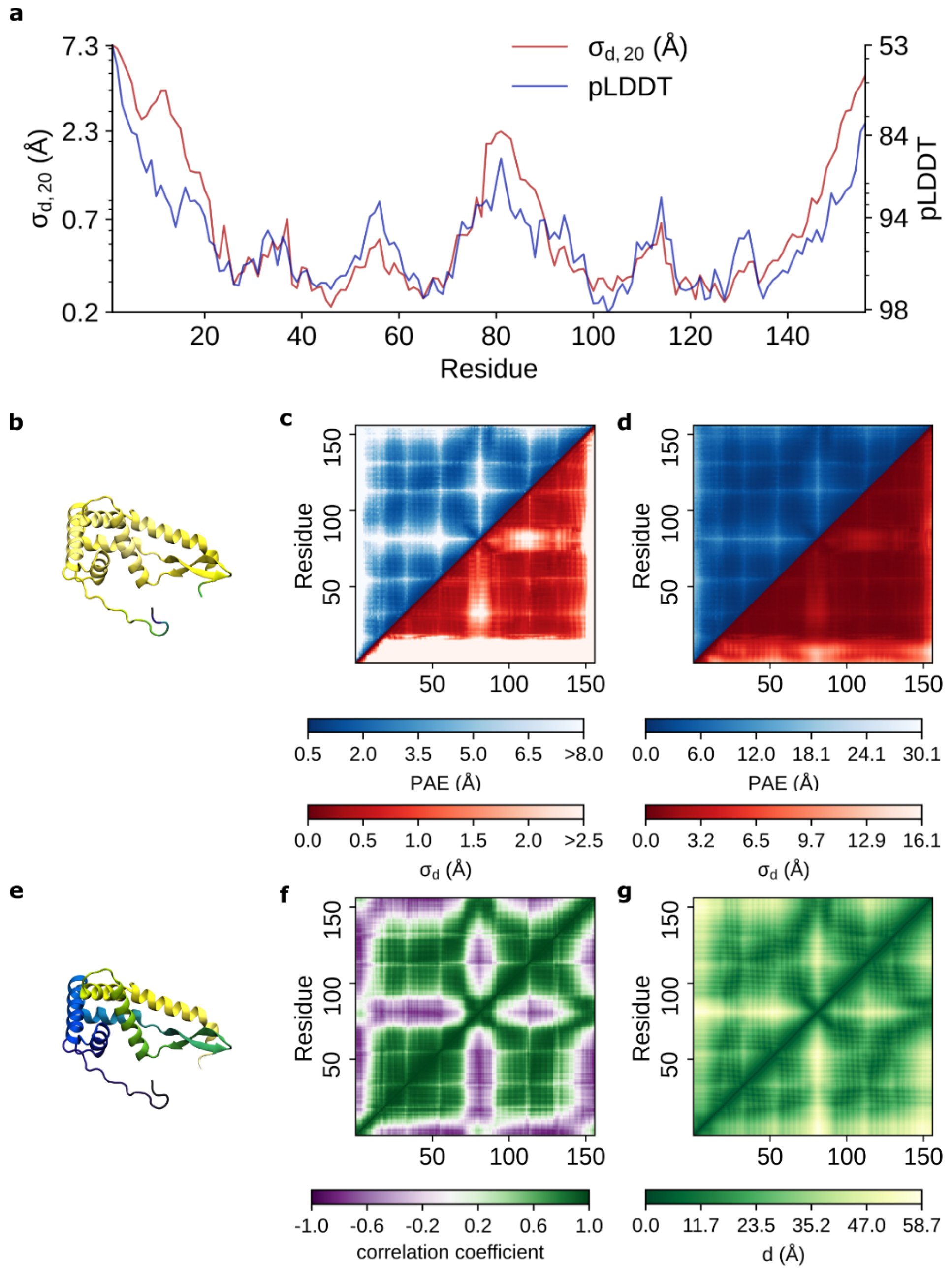

Figure S16: AlphaFold vs. aMD for *30S ribosomal protein S7* (protein 16) **a**) pLDDT scores vs. $\sigma_{d,20}$ values **b**) The protein structure colored based on its pLDDT scores. The dark blue colors correspond to residues with a pLDDT score <= 60, while the white color corresponds to residues with a pLDDT score close to 100. **c**) Comparison between (symmetrized) PAE matrices (blue) against the standard deviation of all C_α distances σ_d (red). The PAE scores range between 0.5 to 8.0 while the σ_d are limited to <2.5 Å. **d**) Comparison between (symmetrized) PAE matrices (blue) against the standard deviation of all C_α distances σ_d (red) for the maximum range. **e**) The protein structure colored based on its residue number. Dark blue colors correspond to low values, and yellows correspond to high values. **f**) Distance correlation matrix obtained from aMD simulations. **g**) Distance matrix obtained from aMD simulation.

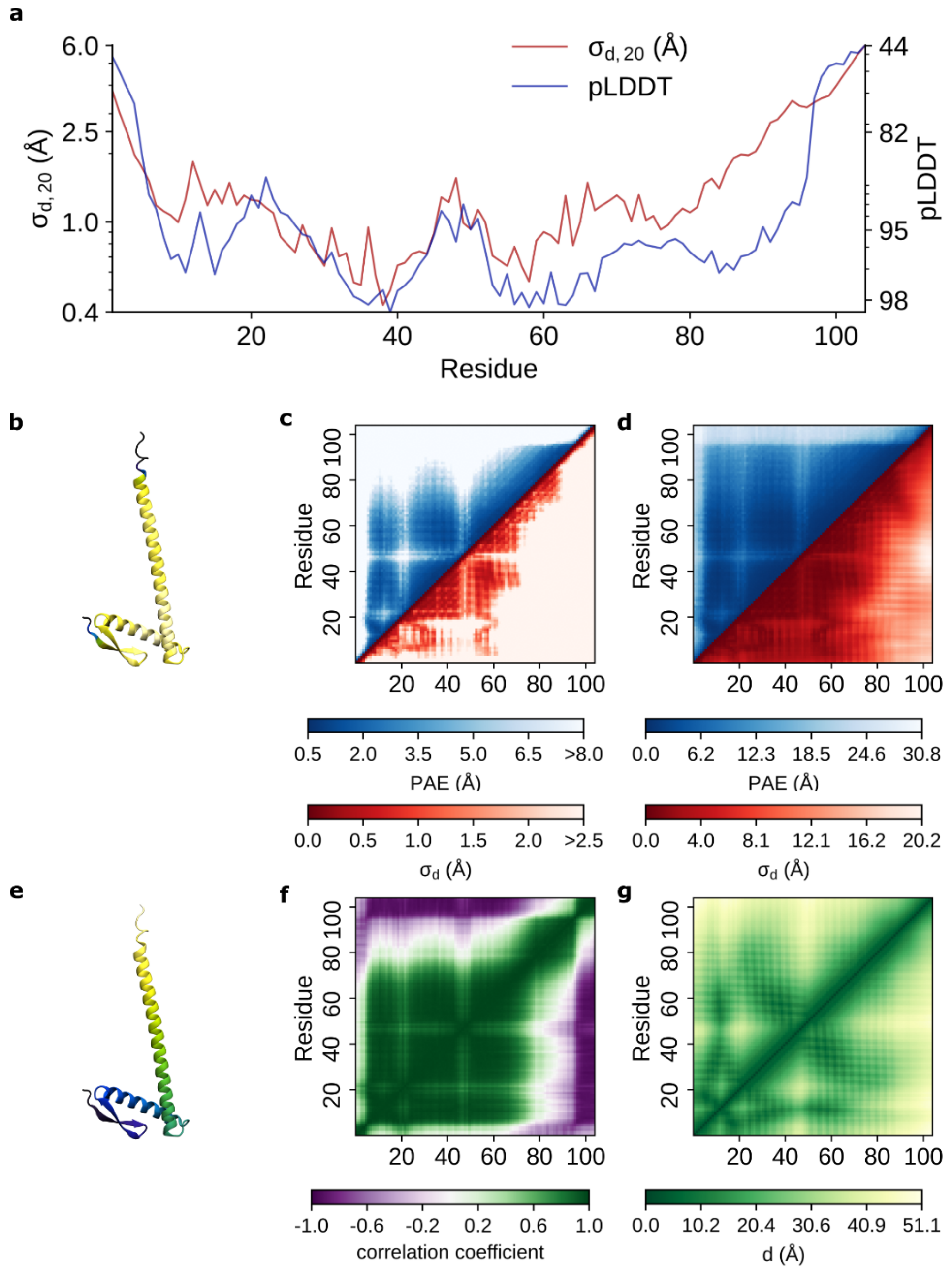

Figure S17: AlphaFold vs. aMD for *cell division protein ZapA* (protein 17) **a**) pLDDT scores vs. $\sigma_{d,20}$ values **b**) The protein structure colored based on its pLDDT scores. The dark blue colors correspond to residues with a pLDDT score <= 60, while the white color corresponds to residues with a pLDDT score close to 100. **c**) Comparison between (symmetrized) PAE matrices (blue) against the standard deviation of all C_α distances σ_d (red). The PAE scores range between 0.5 to 8.0 while the σ_d are limited to <2.5 Å. **d**) Comparison between (symmetrized) PAE matrices (blue) against the standard deviation of all C_α distances σ_d (red) for the maximum range. **e**) The protein structure colored based on its residue number. Dark blue colors correspond to low values, and yellows correspond to high values. **f**) Distance correlation matrix obtained from aMD simulations. **g**) Distance matrix obtained from aMD simulation.

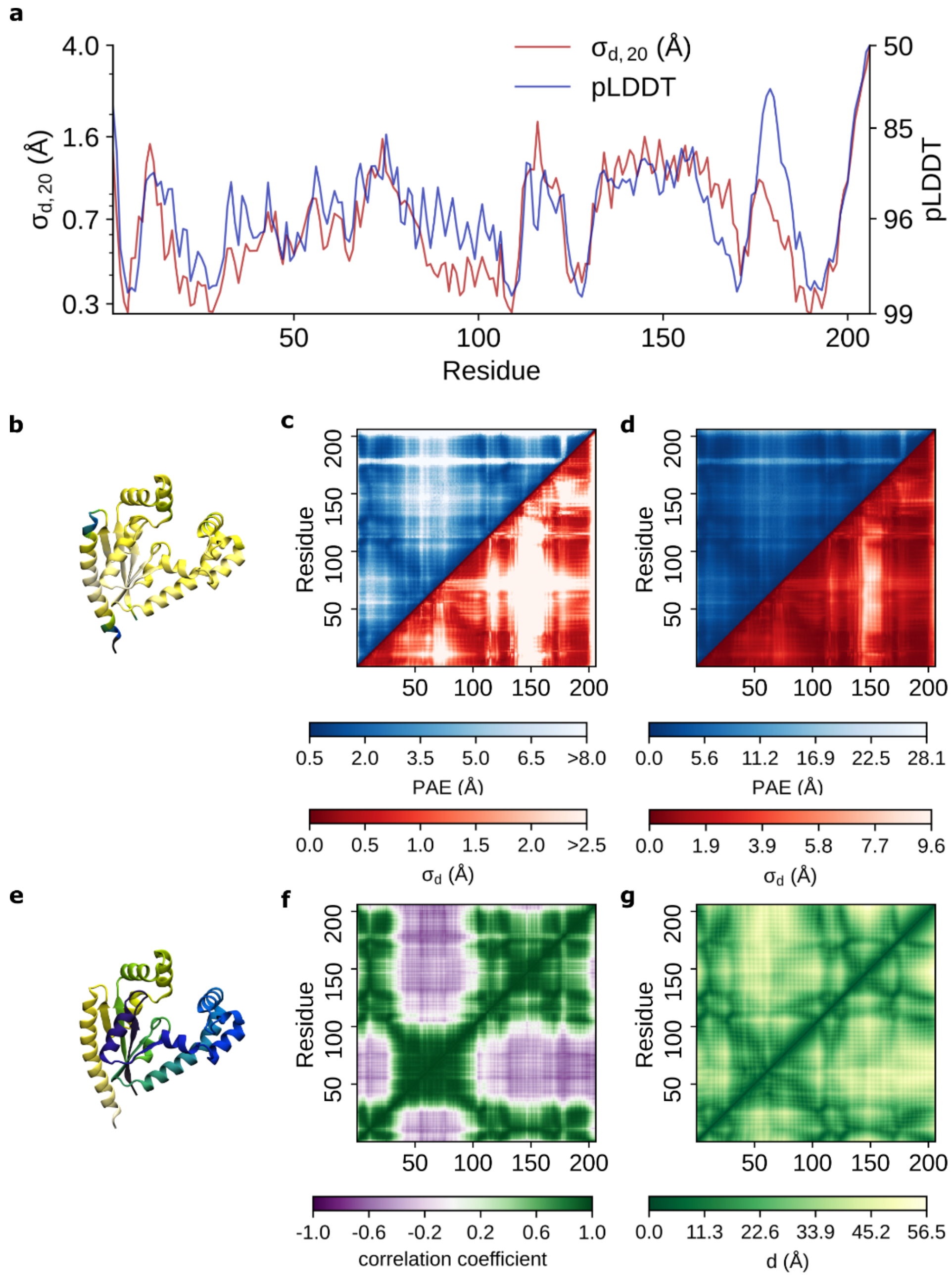

Figure S18: AlphaFold vs. aMD for *dephospho-CoA kinase* (protein 18) **a**) pLDDT scores vs. $\sigma_{d,20}$ values **b**) The protein structure colored based on its pLDDT scores. The dark blue colors correspond to residues with a pLDDT score <= 60, while the white color corresponds to residues with a pLDDT score close to 100. **c**) Comparison between (symmetrized) PAE matrices (blue) against the standard deviation of all C_α distances σ_d (red). The PAE scores range between 0.5 to 8.0 while the σ_d are limited to <2.5 Å. **d**) Comparison between (symmetrized) PAE matrices (blue) against the standard deviation of all C_α distances σ_d (red) for the maximum range. **e**) The protein structure colored based on its residue number. Dark blue colors correspond to low values, and yellows correspond to high values. **f**) Distance correlation matrix obtained from aMD simulations. **g**) Distance matrix obtained from aMD simulation.

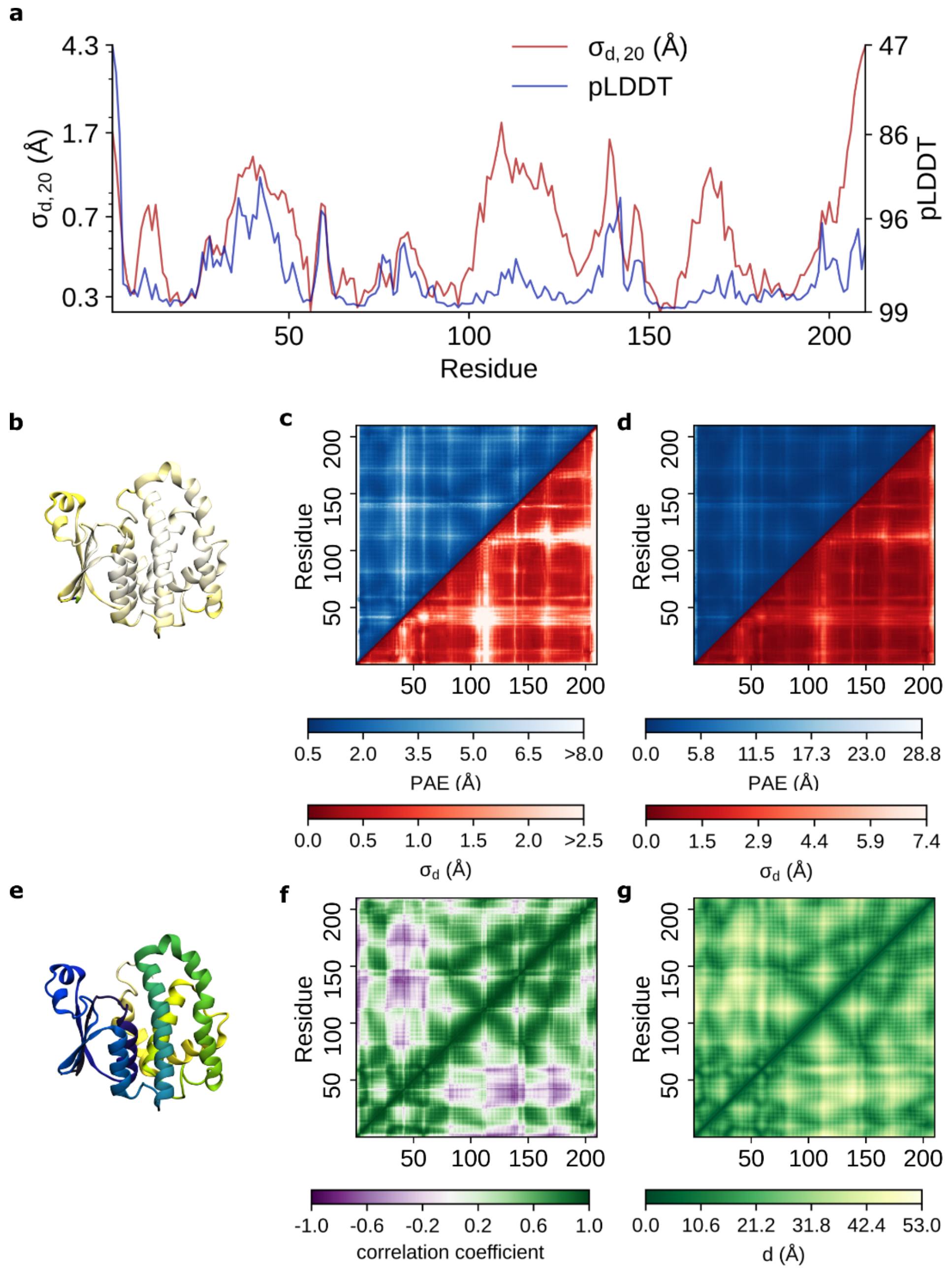

Figure S19: AlphaFold vs. aMD for *glutathione S-transferase P 1* (protein 19) **a**) pLDDT scores vs. $\sigma_{d,20}$ values **b**) The protein structure colored based on its pLDDT scores. The dark blue colors correspond to residues with a pLDDT score <= 60, while the white color corresponds to residues with a pLDDT score close to 100. **c**) Comparison between (symmetrized) PAE matrices (blue) against the standard deviation of all C_α distances σ_d (red). The PAE scores range between 0.5 to 8.0 while the σ_d are limited to <2.5 Å. **d**) Comparison between (symmetrized) PAE matrices (blue) against the standard deviation of all C_α distances σ_d (red) for the maximum range. **e**) The protein structure colored based on its residue number. Dark blue colors correspond to low values, and yellows correspond to high values. **f**) Distance correlation matrix obtained from aMD simulations. **g**) Distance matrix obtained from aMD simulation.

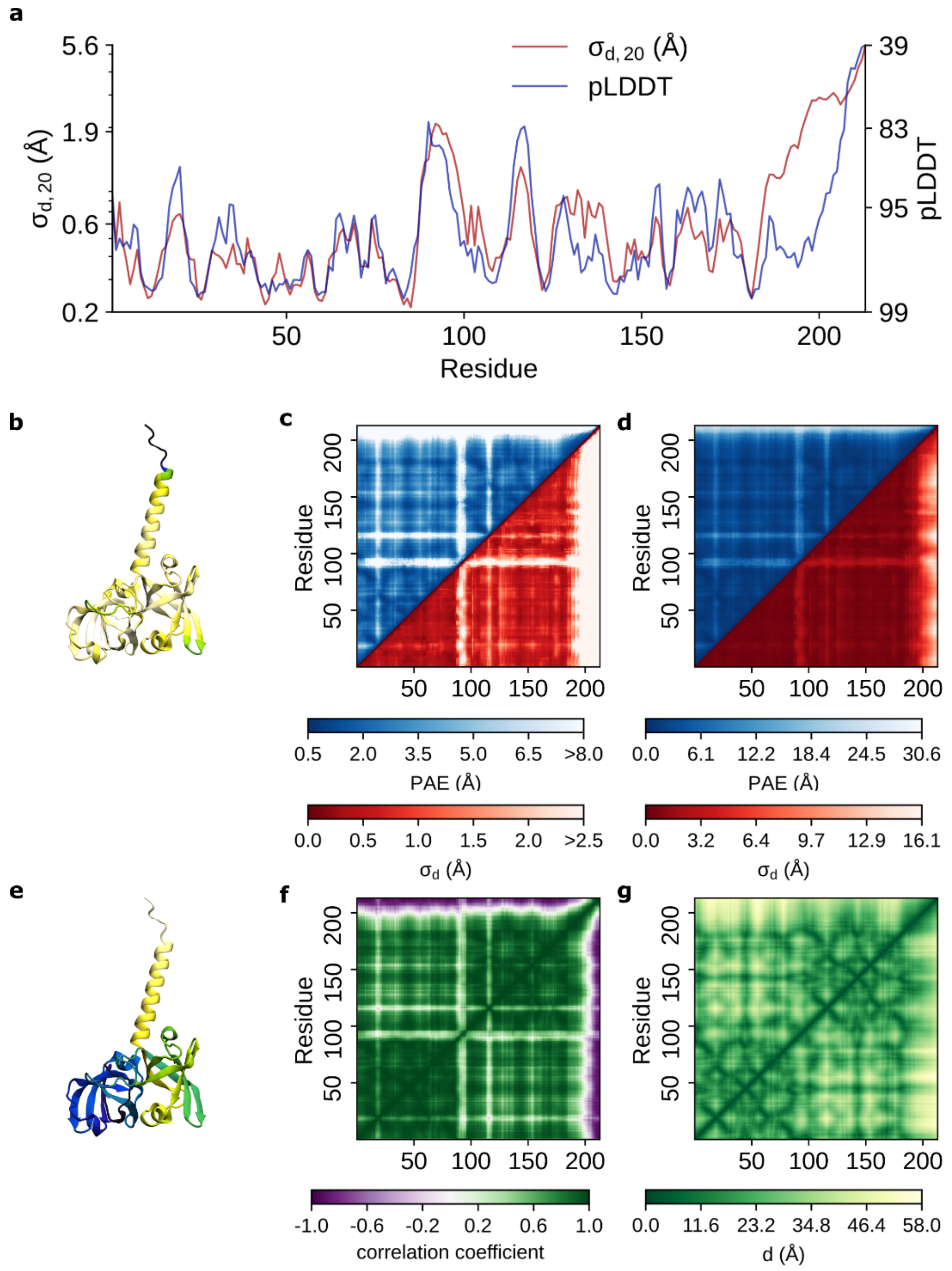

Figure S20: AlphaFold vs. aMD for *riboflavin synthase* (protein 20) **a**) pLDDT scores vs. $\sigma_{d,20}$ values **b**) The protein structure colored based on its pLDDT scores. The dark blue colors correspond to residues with a pLDDT score <= 60, while the white color corresponds to residues with a pLDDT score close to 100. **c**) Comparison between (symmetrized) PAE matrices (blue) against the standard deviation of all C_α distances σ_d (red). The PAE scores range between 0.5 to 8.0 while the σ_d are limited to <2.5 Å. **d**) Comparison between (symmetrized) PAE matrices (blue) against the standard deviation of all C_α distances σ_d (red) for the maximum range. **e**) The protein structure colored based on its residue number. Dark blue colors correspond to low values, and yellows correspond to high values. **f**) Distance correlation matrix obtained from aMD simulations. **g**) Distance matrix obtained from aMD simulation.

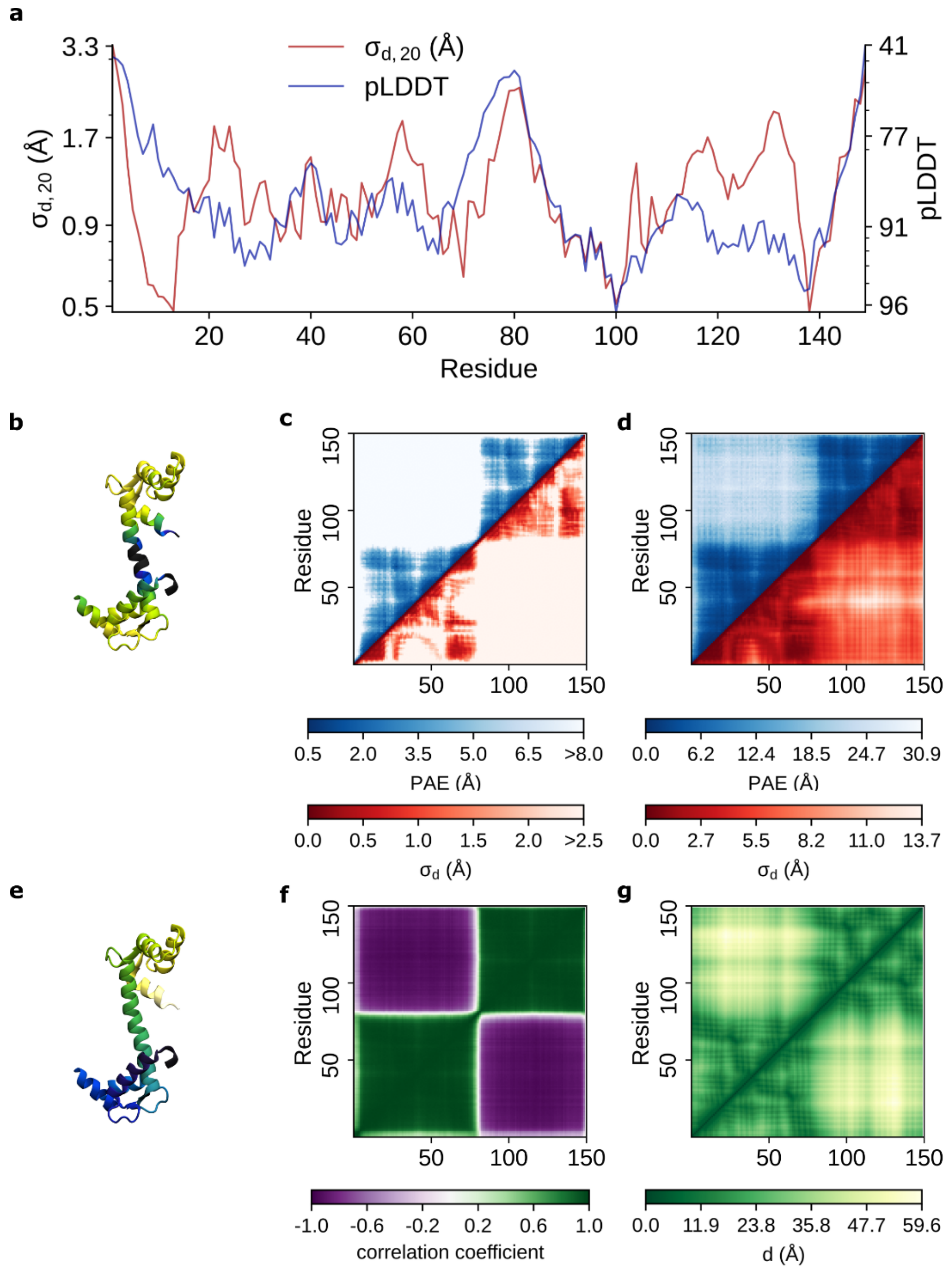

Figure S21: AlphaFold vs. aMD for *calmodulin* (protein 21) **a**) pLDDT scores vs. $\sigma_{d,20}$ values **b**) The protein structure colored based on its pLDDT scores. The dark blue colors correspond to residues with a pLDDT score <= 60, while the white color corresponds to residues with a pLDDT score close to 100. **c**) Comparison between (symmetrized) PAE matrices (blue) against the standard deviation of all C_α distances σ_d (red). The PAE scores range between 0.5 to 8.0 while the σ_d are limited to <2.5 Å. **d**) Comparison between (symmetrized) PAE matrices (blue) against the standard deviation of all C_α distances σ_d (red) for the maximum range. **e**) The protein structure colored based on its residue number. Dark blue colors correspond to low values, and yellows correspond to high values. **f**) Distance correlation matrix obtained from aMD simulations. **g**) Distance matrix obtained from aMD simulation.

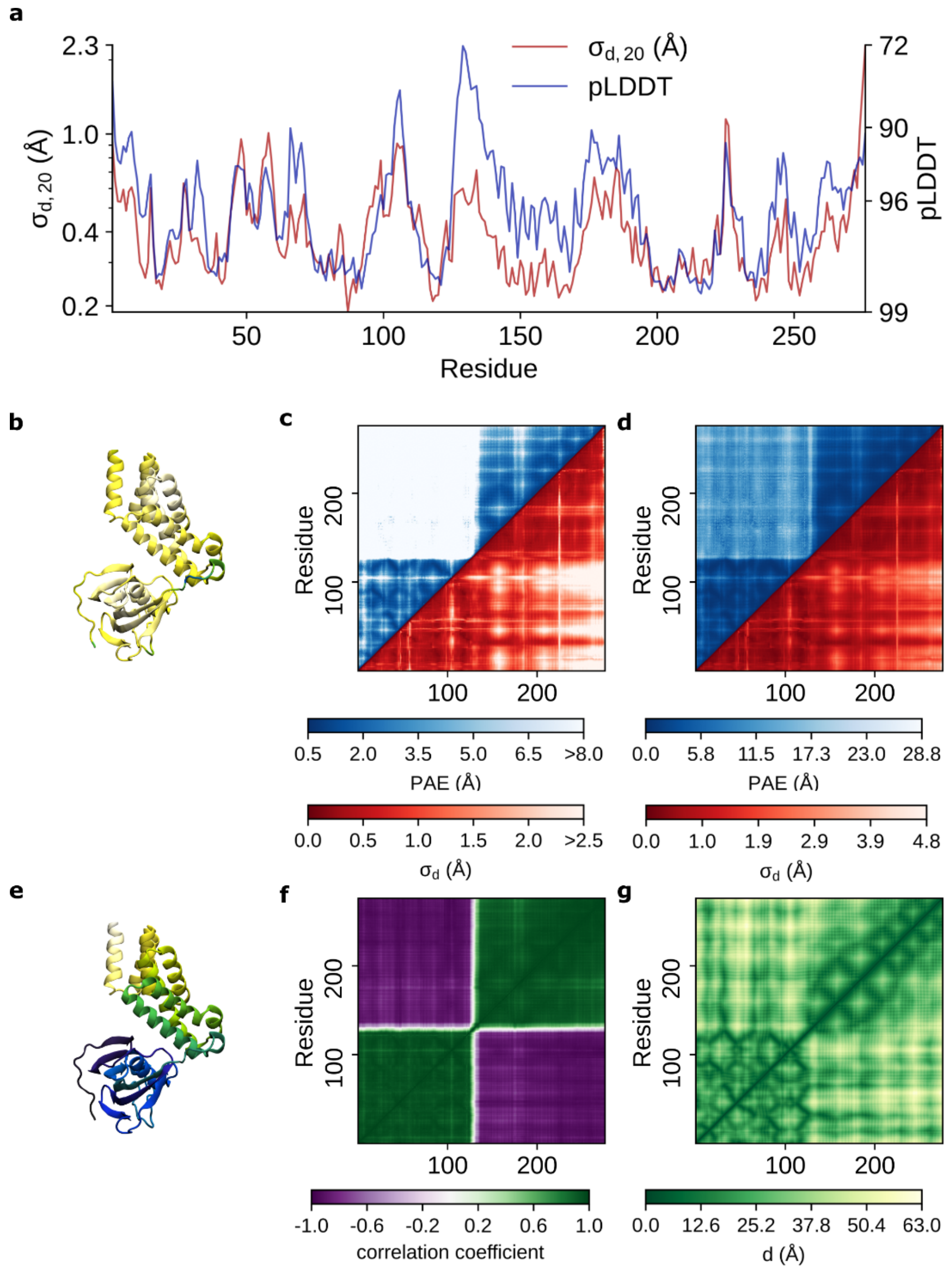

Figure S22: AlphaFold vs. aMD for *peptidyl-prolyl cis-trans isomerase FKBP42* (protein 22) **a**) pLDDT scores vs. $\sigma_{d,20}$ values **b**) The protein structure colored based on its pLDDT scores. The dark blue colors correspond to residues with a pLDDT score <= 60, while the white color corresponds to residues with a pLDDT score close to 100. **c**) Comparison between (symmetrized) PAE matrices (blue) against the standard deviation of all C_α distances σ_d (red). The PAE scores range between 0.5 to 8.0 while the σ_d are limited to <2.5 Å. **d**) Comparison between (symmetrized) PAE matrices (blue) against the standard deviation of all C_α distances σ_d (red) for the maximum range. **e**) The protein structure colored based on its residue number. Dark blue colors correspond to low values, and yellows correspond to high values. **f**) Distance correlation matrix obtained from aMD simulations. **g**) Distance matrix obtained from aMD simulation.

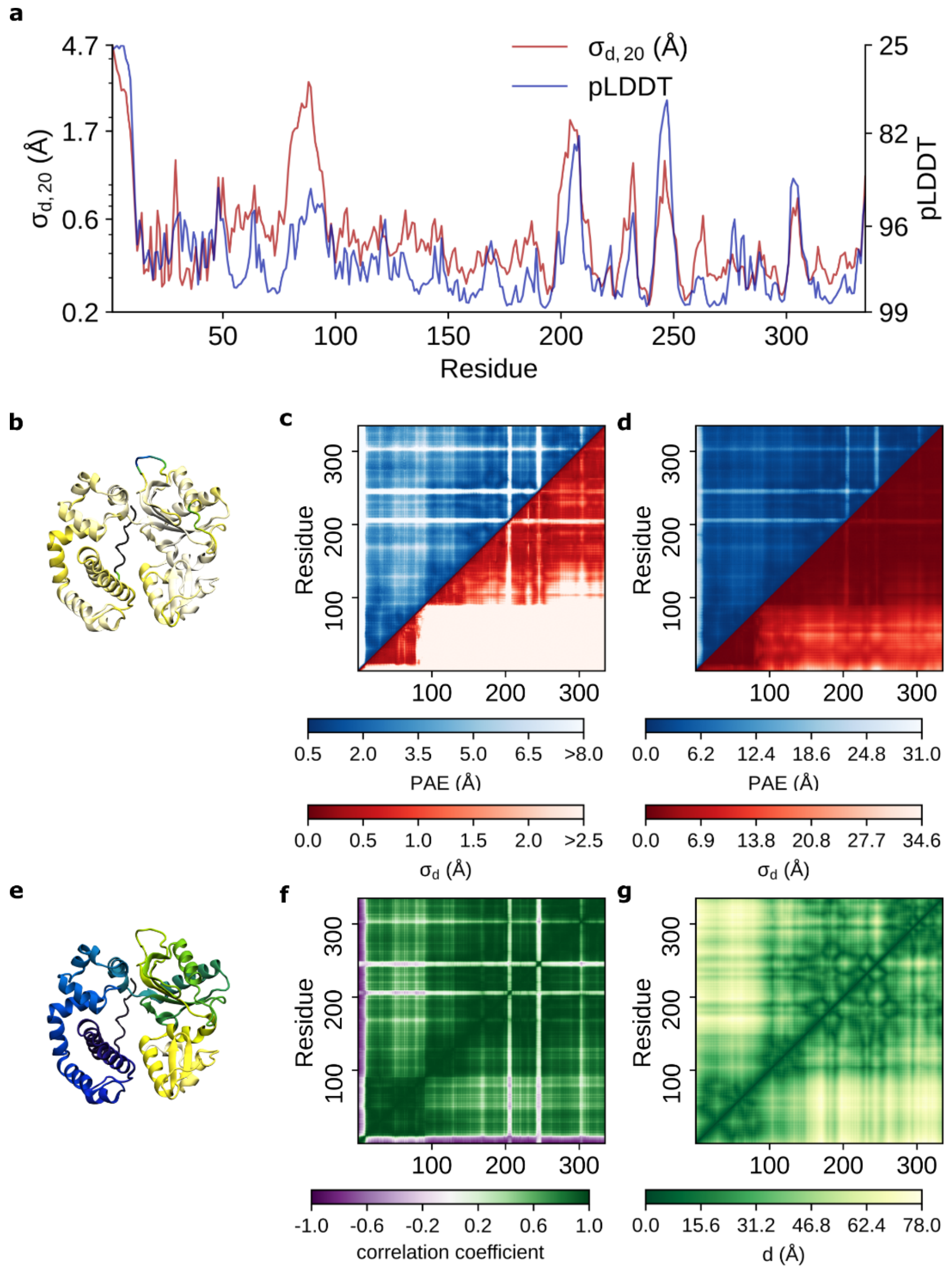

Figure S23: AlphaFold vs. aMD for *DNA polymerase beta* (protein 23) **a**) pLDDT scores vs. $\sigma_{d,20}$ values **b**) The protein structure colored based on its pLDDT scores. The dark blue colors correspond to residues with a pLDDT score <= 60, while the white color corresponds to residues with a pLDDT score close to 100. **c**) Comparison between (symmetrized) PAE matrices (blue) against the standard deviation of all C_α distances σ_d (red). The PAE scores range between 0.5 to 8.0 while the σ_d are limited to <2.5 Å. **d**) Comparison between (symmetrized) PAE matrices (blue) against the standard deviation of all C_α distances σ_d (red) for the maximum range. **e**) The protein structure colored based on its residue number. Dark blue colors correspond to low values, and yellows correspond to high values. **f**) Distance correlation matrix obtained from aMD simulations. **g**) Distance matrix obtained from aMD simulation.

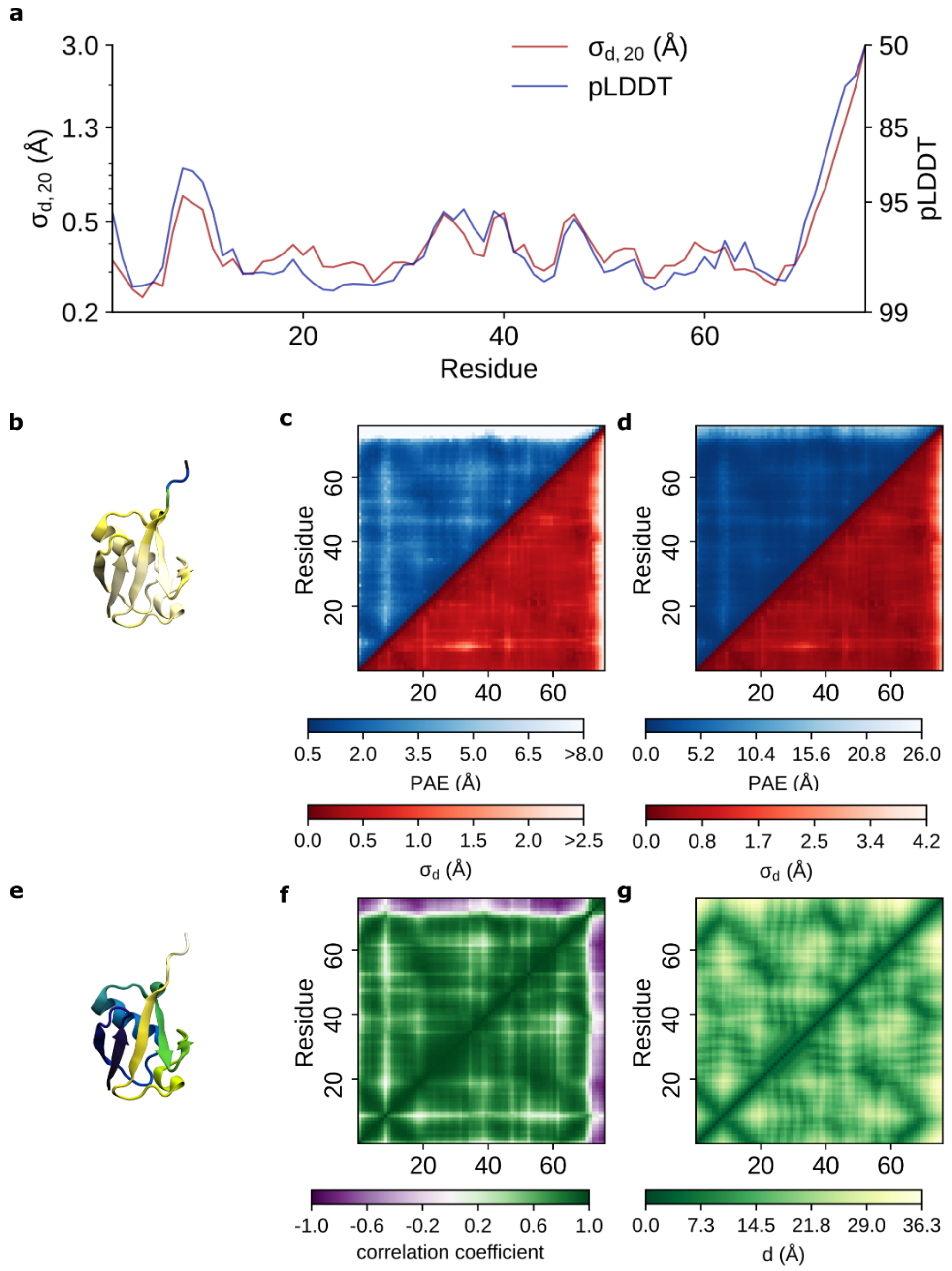

Figure S24: AlphaFold vs. aMD for *ubiquitin* (protein 24) **a**) pLDDT scores vs. $\sigma_{d,20}$ values **b**) The protein structure colored based on its pLDDT scores. The dark blue colors correspond to residues with a pLDDT score <= 60, while the white color corresponds to residues with a pLDDT score close to 100. **c**) Comparison between (symmetrized) PAE matrices (blue) against the standard deviation of all C_α distances σ_d (red). The PAE scores range between 0.5 to 8.0 while the σ_d are limited to <2.5 Å. **d**) Comparison between (symmetrized) PAE matrices (blue) against the standard deviation of all C_α distances σ_d (red) for the maximum range. **e**) The protein structure colored based on its residue number. Dark blue colors correspond to low values, and yellows correspond to high values. **f**) Distance correlation matrix obtained from aMD simulations. **g**) Distance matrix obtained from aMD simulation.

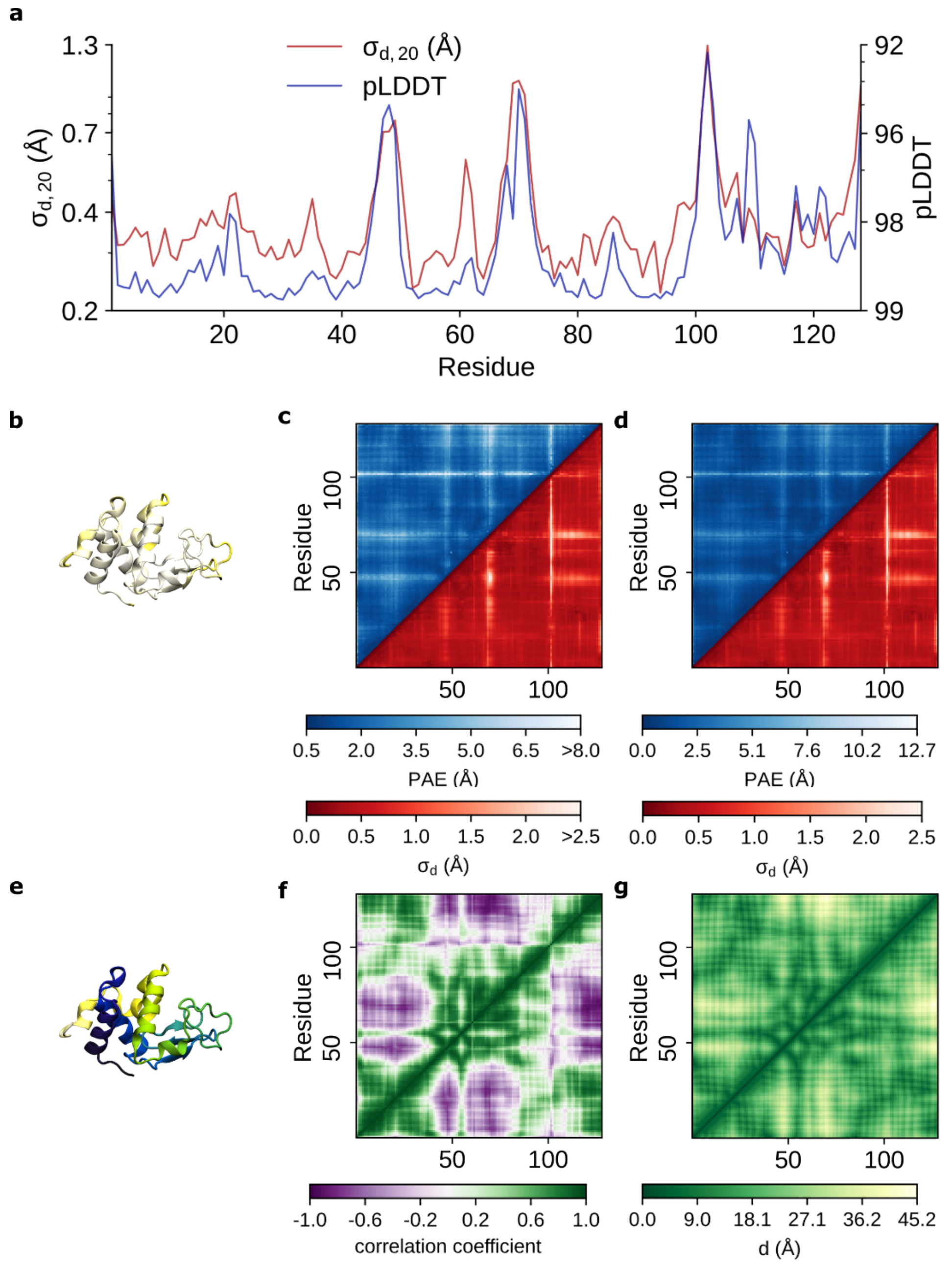

Figure S25: AlphaFold vs. aMD for *lysozyme C* (protein 25) **a**) pLDDT scores vs. $\sigma_{d,20}$ values **b**) The protein structure colored based on its pLDDT scores. The dark blue colors correspond to residues with a pLDDT score <= 60, while the white color corresponds to residues with a pLDDT score close to 100. **c**) Comparison between (symmetrized) PAE matrices (blue) against the standard deviation of all C_α distances σ_d (red). The PAE scores range between 0.5 to 8.0 while the σ_d are limited to <2.5 Å. **d**) Comparison between (symmetrized) PAE matrices (blue) against the standard deviation of all C_α distances σ_d (red) for the maximum range. **e**) The protein structure colored based on its residue number. Dark blue colors correspond to low values, and yellows correspond to high values. **f**) Distance correlation matrix obtained from aMD simulations. **g**) Distance matrix obtained from aMD simulation.

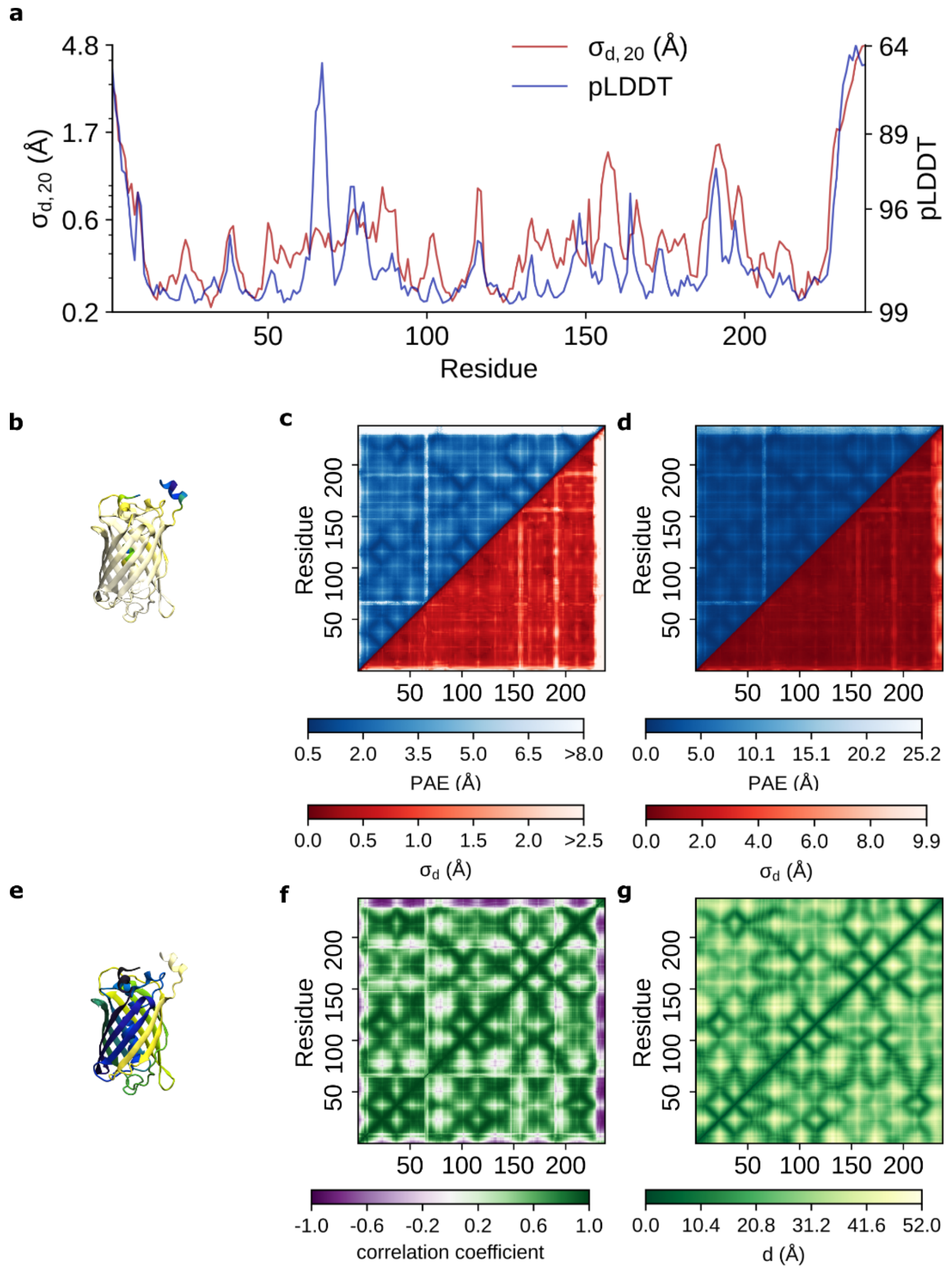

Figure S26: AlphaFold vs. aMD for *green fluorescent protein* (protein 26) **a**) pLDDT scores vs. $\sigma_{d,20}$ values **b**) The protein structure colored based on its pLDDT scores. The dark blue colors correspond to residues with a pLDDT score <= 60, while the white color corresponds to residues with a pLDDT score close to 100. **c**) Comparison between (symmetrized) PAE matrices (blue) against the standard deviation of all C_α distances σ_d (red). The PAE scores range between 0.5 to 8.0 while the σ_d are limited to <2.5 Å. **d**) Comparison between (symmetrized) PAE matrices (blue) against the standard deviation of all C_α distances σ_d (red) for the maximum range. **e**) The protein structure colored based on its residue number. Dark blue colors correspond to low values, and yellows correspond to high values. **f**) Distance correlation matrix obtained from aMD simulations. **g**) Distance matrix obtained from aMD simulation.

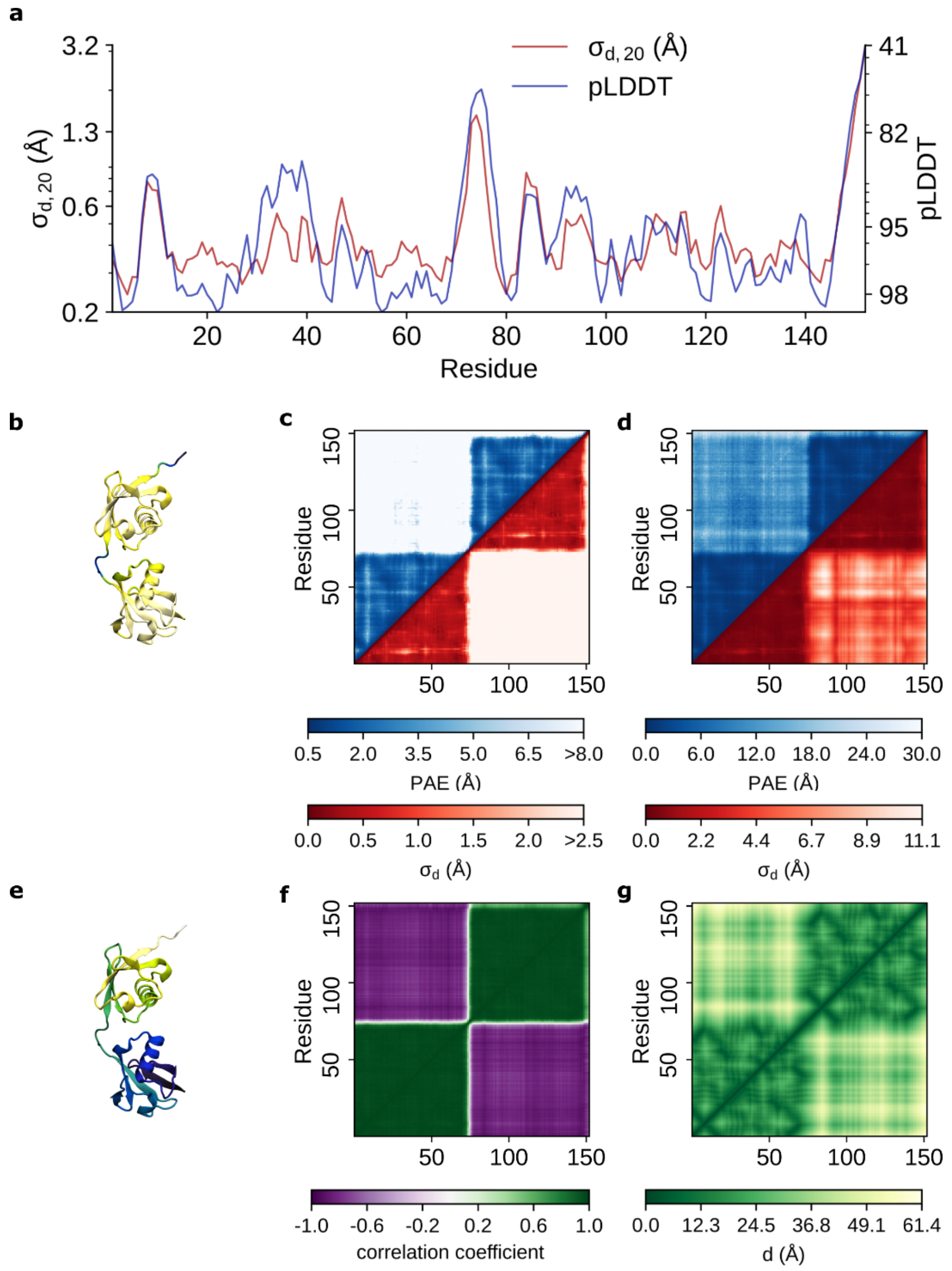

Figure S27: AlphaFold vs. aMD for *linear diubiquitin* (protein 27) **a**) pLDDT scores vs. $\sigma_{d,20}$ values **b**) The protein structure colored based on its pLDDT scores. The dark blue colors correspond to residues with a pLDDT score <= 60, while the white color corresponds to residues with a pLDDT score close to 100. **c**) Comparison between (symmetrized) PAE matrices (blue) against the standard deviation of all C_α distances σ_d (red). The PAE scores range between 0.5 to 8.0 while the σ_d are limited to <2.5 Å. **d**) Comparison between (symmetrized) PAE matrices (blue) against the standard deviation of all C_α distances σ_d (red) for the maximum range. **e**) The protein structure colored based on its residue number. Dark blue colors correspond to low values, and yellows correspond to high values. **f**) Distance correlation matrix obtained from aMD simulations. **g**) Distance matrix obtained from aMD simulation.

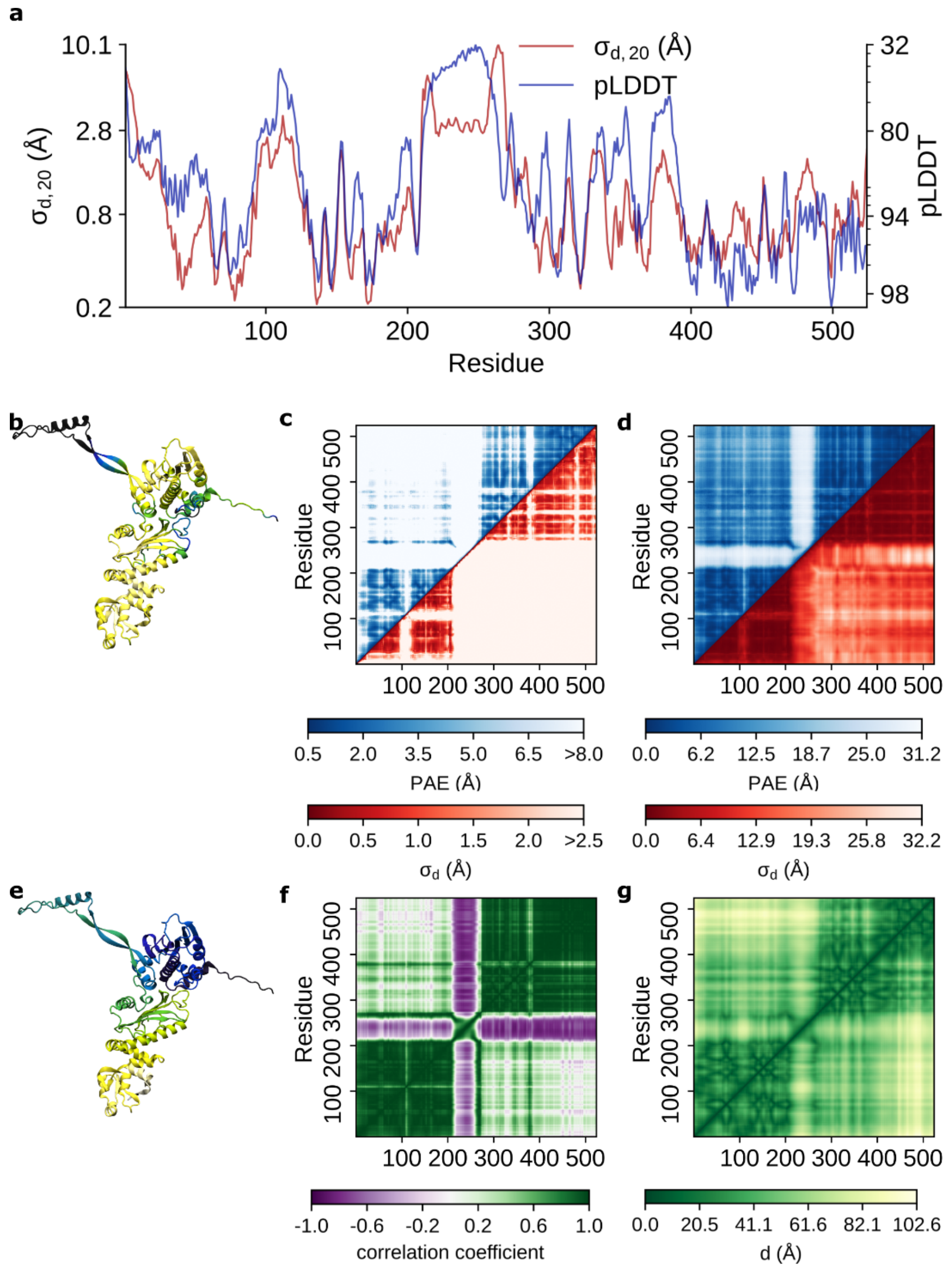

Figure S28: AlphaFold vs. aMD for *ATP-dependent molecular chaperon HSP82* (NTD-MD only) (protein 28) **a**) pLDDT scores vs. $\sigma_{d,20}$ values **b**) The protein structure colored based on its pLDDT scores. The dark blue colors correspond to residues with a pLDDT score <= 60, while the white color corresponds to residues with a pLDDT score close to 100. **c**) Comparison between (symmetrized) PAE matrices (blue) against the standard deviation of all C_α distances σ_d (red). The PAE scores range between 0.5 to 8.0 while the σ_d are limited to <2.5 Å. **d**) Comparison between (symmetrized) PAE matrices (blue) against the standard deviation of all C_α distances σ_d (red) for the maximum range. **e**) The protein structure colored based on its residue number. Dark blue colors correspond to low values, and yellows correspond to high values. **f**) Distance correlation matrix obtained from aMD simulations. **g**) Distance matrix obtained from aMD simulation.

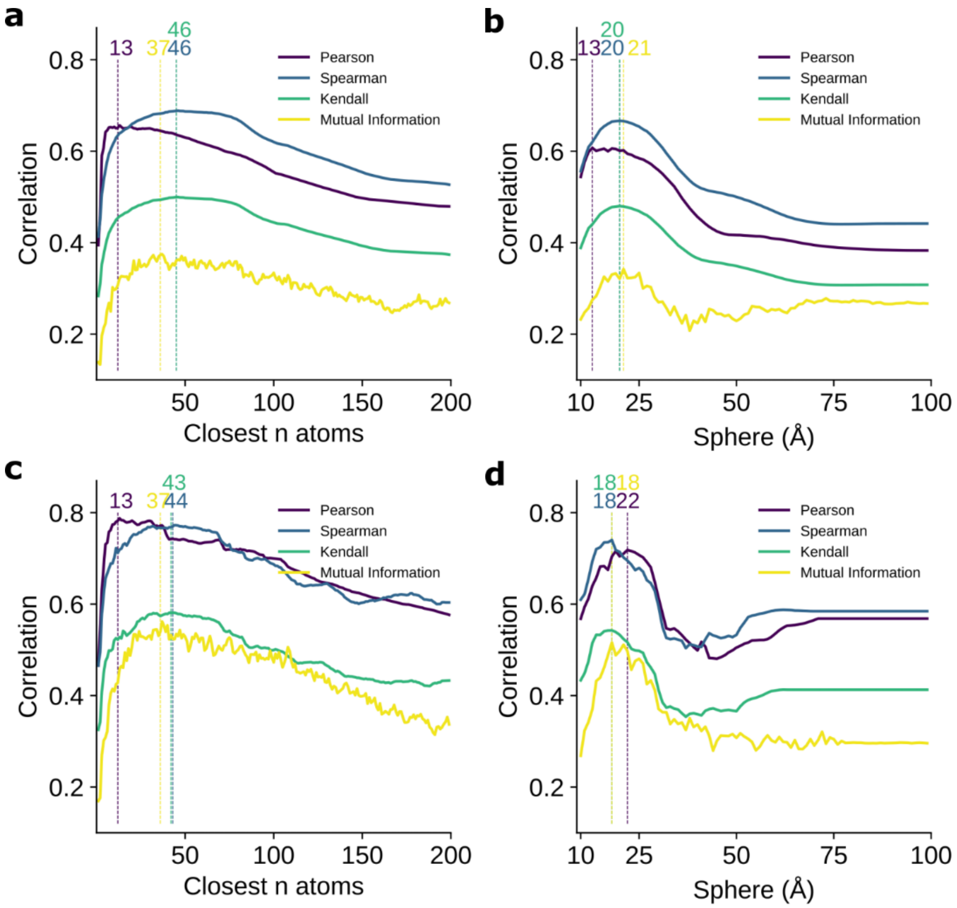

Figure S29: Comparison of linear and non-linear correlations between pLDDT and $\sigma_{d,n}$ **a**) Dependency of the Pearson, Spearman's rank, Kendall rank, and Mutual Information correlation coefficients from the number (*n*) of closest atoms included in $\sigma_{d,n}$ calculation (pLDDT and $\sigma_{d,n}$ data combined across all systems). **b**) Dependency of correlation coefficients including all C_α in a given sphere to calculate $\sigma_{d,n}$ (pLDDT and $\sigma_{d,n}$ data combined across all systems). **c**) Median correlation coefficients across all 28 systems vs. the number (*n*) of closest atoms included in $\sigma_{d,n}$ calculation **d**) Median correlation coefficients across all 28 systems vs. sphere size for $\sigma_{d,n}$ calculation.

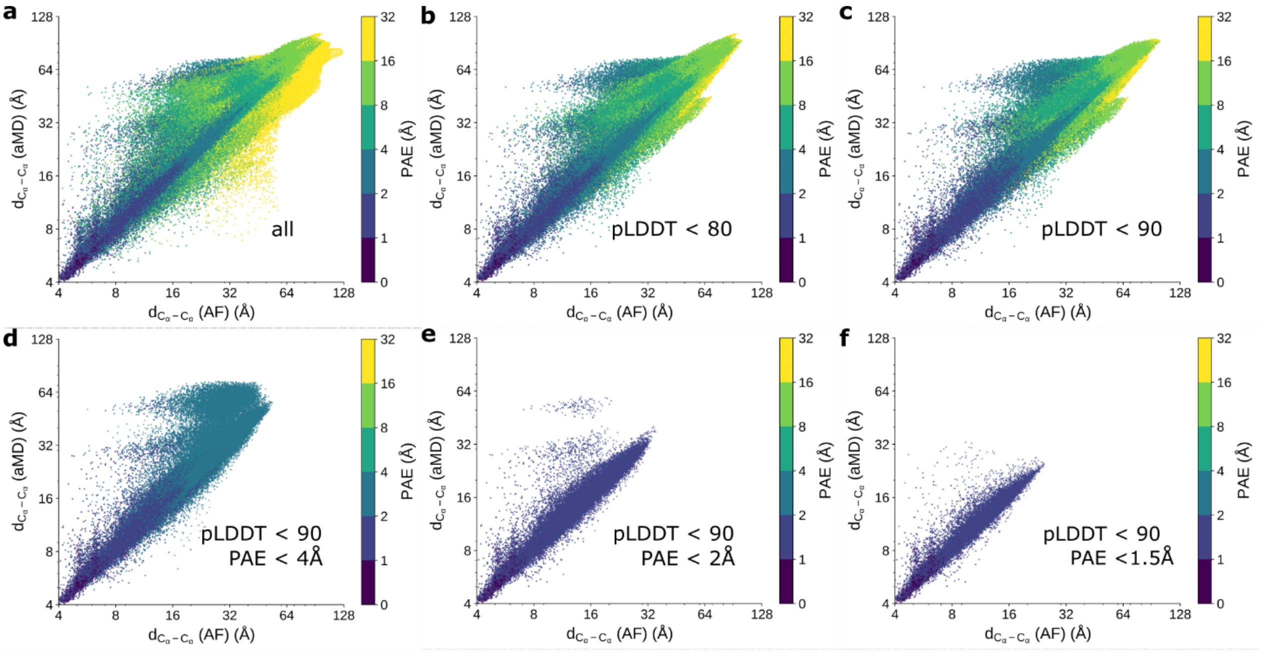

Figure S30: Protein distances predicted by AlphaFold vs. aMD distances. The figure shows scatter plots combined for all systems. Subfigures **a-f**) show the scatter plots for different subsets defined by pLDDT and PAE thresholds. Scatter plots for **a**) all distances, **b**) pLDDT < 80, **c**) pLDDT < 90, **d**) pLDDT < 90 and PAE < 4 Å, **e**) pLDDT < 90 Å and PAE < 2 Å, **f**) pLDDT < 90 and PAE < 1.5 Å. Lower thresholds increase the agreement between AlphaFold and aMD distances.

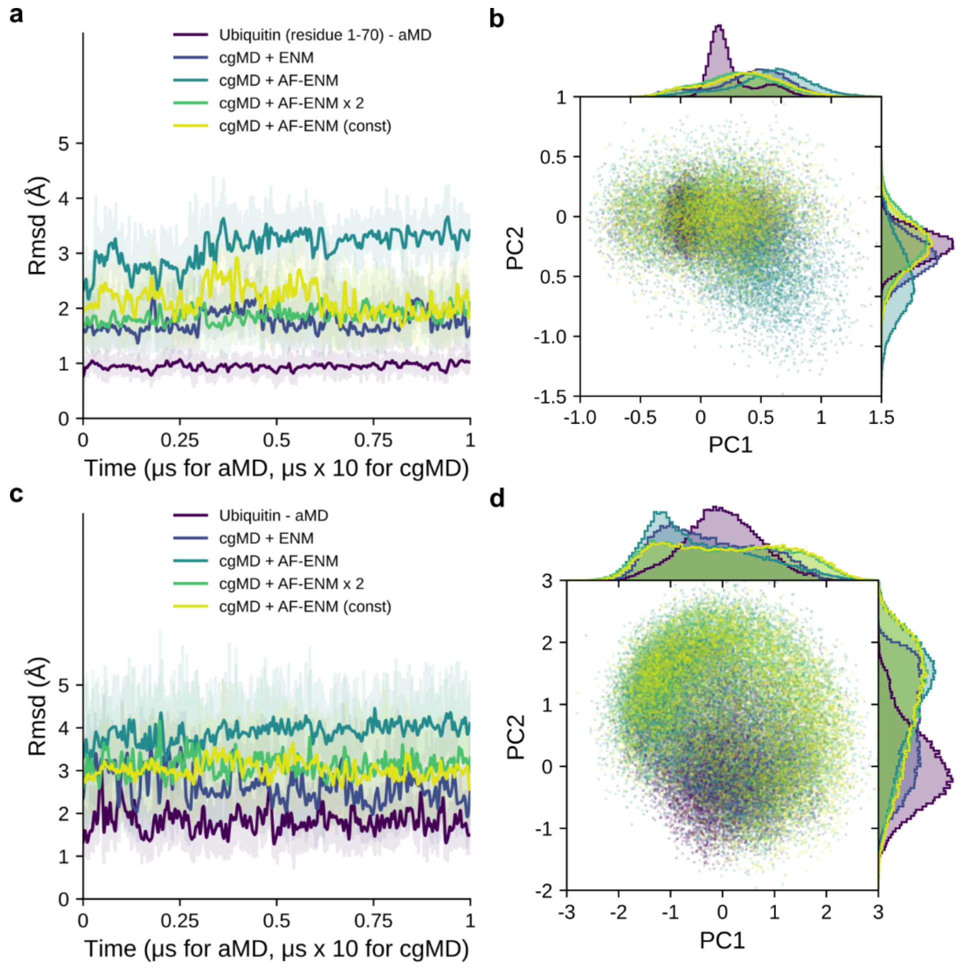

Figure S31: Ubiquitin (protein 24) global dynamics. **a,c**) RSMD of the aMD and cgMD simulations for (a) residue 1 - 70 or (c) complete system. **b,d**) Principal component analysis for aMD and cgMD simulations for (b) residue 1 - 70 or (d) complete system. The cgMD trajectories are projected on the aMD-derived first (PC1) and second (PC2) principal components.

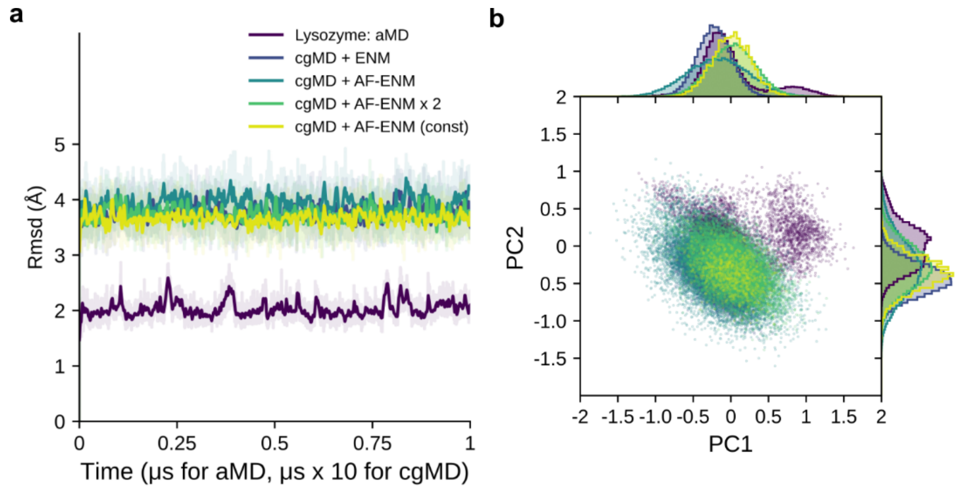

Figure S32: Lysozyme C (protein 25) global dynamics. **a**) RSMD of the aMD and cgMD simulations. **b**) Principal component analysis of aMD and cgMD simulations. The cgMD trajectories are projected on the aMD-derived first (PC1) and second (PC2) principal components.

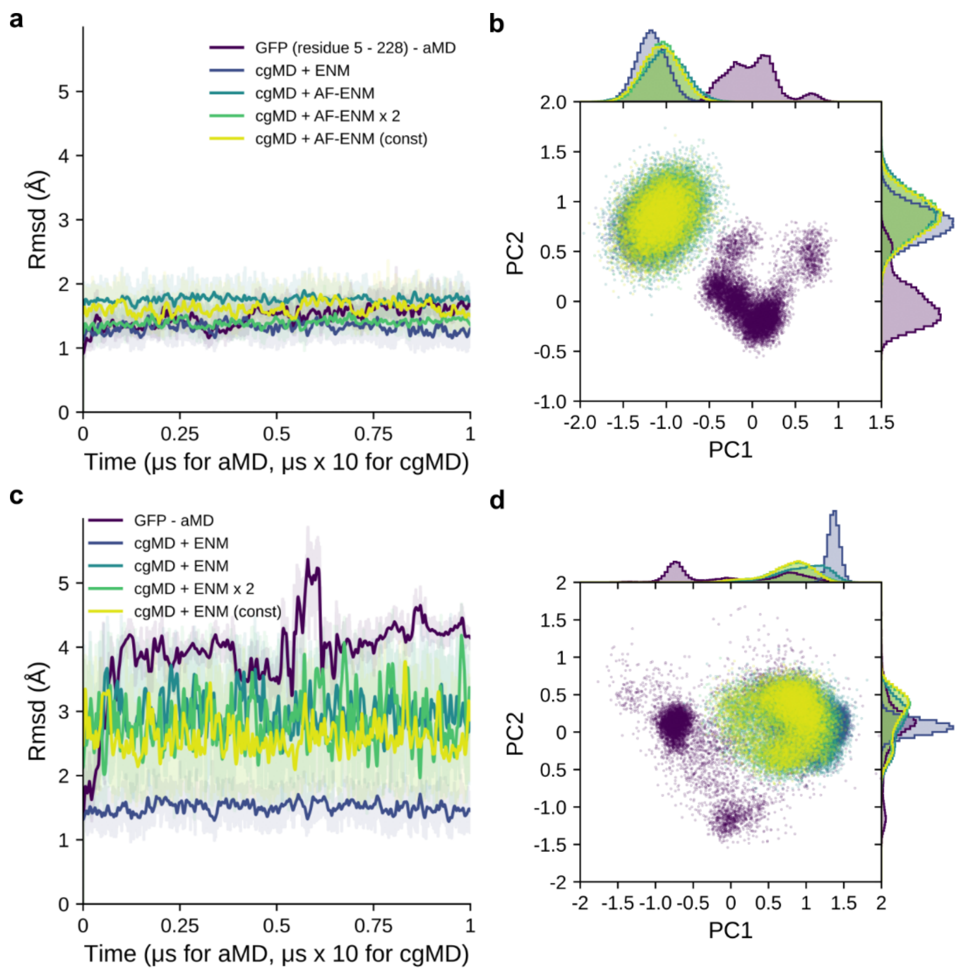

Figure S33: Green fluorescent protein (protein 26) global dynamics. **a,c**) RSMD of the aMD and cgMD simulations for (a) residue 5 - 228 or (c) complete system. **b,d**) Principal component analysis for aMD and cgMD simulations for (b) residue5 - 228 or (d) complete system. The cgMD trajectories are projected on the aMD-derived first (PC1) and second (PC2) principal components.

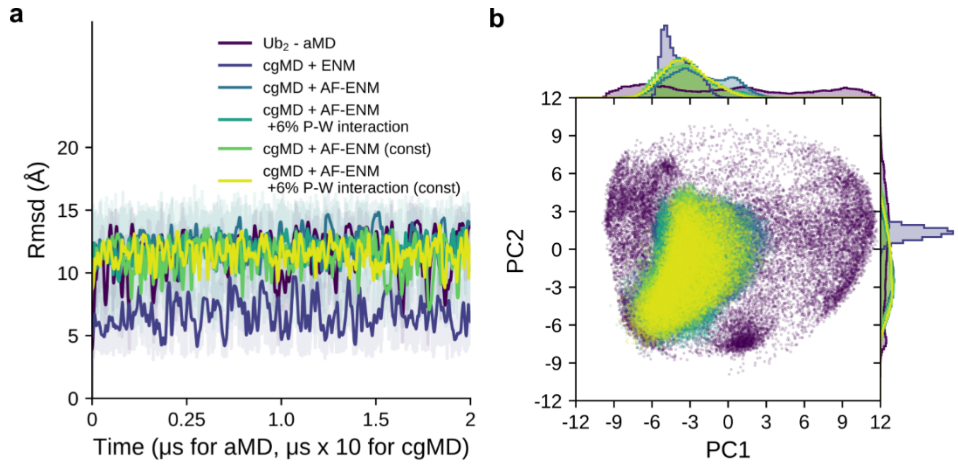

Figure S34: Linear di-ubiquitin (protein 27) global dynamics. **a**) RSMD of the aMD and cgMD simulations. **b**) Principal component analysis of aMD and cgMD simulations. The cgMD trajectories are projected on the aMD-derived first (PC1) and second (PC2) principal components. The cgMD trajectories are projected on the aMD-derived first (PC1) and second (PC2) principal components.

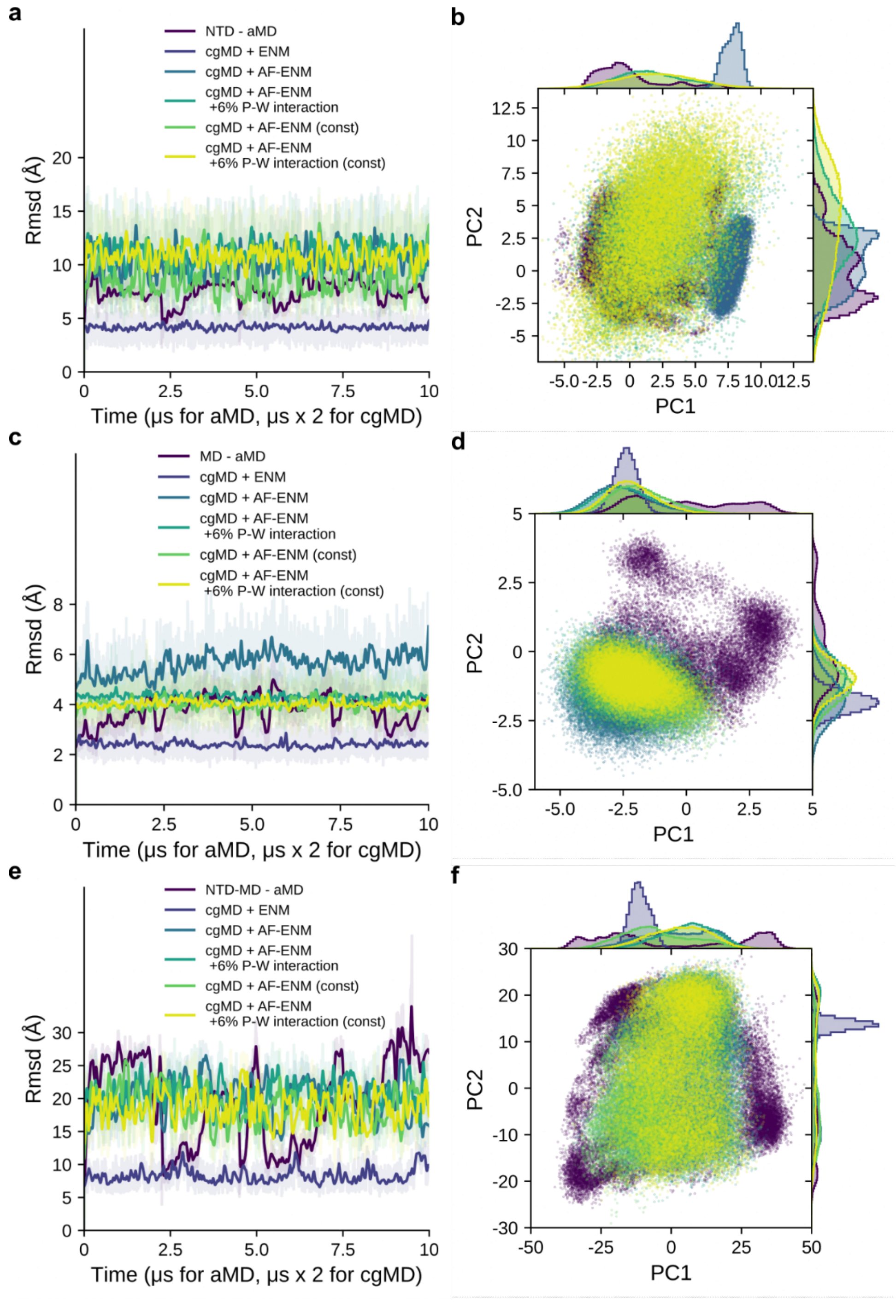

Figure S35: NTD-MD domain of Hsp90 (protein 28) global dynamics. **a,c,e**) RSMD of the aMD and cgMD simulations for (a) the N-terminal domain (NTD, residue 1 – 210), (c) middle domain (MD, residue 275 – 524) or (e) complete NTD-MD system (residue 1 - 524). . **b,d,f**) Principal component analysis for aMD and cgMD simulations for (b) NTD, (d) MD or (f) NTD-MD. The cgMD trajectories are projected on the aMD-derived first (PC1) and second (PC2) principal components.

Table S1: Summary of all simulated proteins.

protein	Name	UniProt	Amino acids	Amino acid range	Simulation length [μs]	System size
1	Arginine repressor	O31408	149	1 - 149	2	87,444
2	WW domain-binding protein 4	O75554	75	122 - 196	2	84,175
3	Nuclear factor of activated T-cells, cytoplasmic 2	Q13469	280	399 - 678	2	207,648
4	Killer cell immunoglobulin-like receptor 2DL1	P43626	200	22 - 221	2	96,392
5	Transcription inhibitor protein Gfh1	Q8VQD7	157	1 - 157	2	110,503
6	Protein windbeutel	O44342	223	21 - 257	2	131,963
7	Sorcin	P30626	198	1 - 198	2	112,655
8	GTPase Era	P06616	301	1 - 301	2	96,207
9	Elongation factor Ts	P43895	196	1 - 196	2	83,255
10	14-3-3 protein beta/alpha	P31946	246	1 - 246	2	162,596
11	Chloride intracellular channel protein 1	O00299	241	1 - 241	2	95,819
12	Gamma-crystallin B	P02526	175	1 - 175	2	61,795
13	Transposon Tn7 transposition protein TnsA	P13988	273	1 - 273	2	100,299
14	NAD(P)H-flavin reductase	P0AEN1	233	1 - 233	2	64,350
15	Cyclin-dependent kinase 2	P24941	298	1 - 298	2	84,322
16	30S ribosomal protein S7	P17291	156	1 - 156	2	68,090
17	Cell division protein ZapA	Q9HTW3	104	1 - 104	2	161,499
18	Dephospho-CoA kinase	P0A6I9	206	1 - 206	2	70,273
19	Glutathione S-transferase P 1	P19157	210	1 - 210	2	63,788
20	Riboflavin synthase	P0AFU8	213	1 - 213	2	119,495
21	Calmodulin	O16305	149	1 - 149	2	91,499
22	Peptidyl-prolyl cis-trans isomerase FKBP42	Q9LDC0	276	35 - 310	2	96,889
23	DNA polymerase beta	P06766	335	1 - 335	2	98,518
24	Ubiquitin	P0CG48	76	1 - 76	1	38,843
25	Lysozyme C	P00698	128	1 - 128	1	49,023
26	Green fluorescent protein	P42212	238	1 - 238	1	68,624
27	Linear diubiquitin	P0CG48	152	1 - 152	2	92,946
28	ATP-dependent molecular chaperon Hsp82 NTD-MD construct	P02829	524	1 - 524	10.5	542,474

Table S2: Comparison of pLDDT scores across all investigated systems.

protein	*R*	Intercept	Slope	$pLDDT_{min}$	$pLDDT_{max}$	$pLDDT_{median}$
1	0.78	101.40	-15.71	50.44	98.7	96.28
2	0.94	96.68	-16.51	41.88	93.4	86.92
3	0.82	101.83	-17.68	52.21	98.31	95.23
4	0.44	98.29	-7.17	50.92	98.53	95.76
5	0.88	101.77	-19.13	49.45	98.58	93.40
6	0.90	99.58	-8.43	40.73	98.63	95.55
7	0.89	102.11	-16.7	32.44	96.50	92.64
8	0.76	100.11	-20.14	44.39	98.37	92.10
9	0.28	97.67	-1.29	79.10	98.87	97.63
10	0.96	102.41	-13.37	32.90	98.88	97.87
11	0.79	103.07	-10.78	41.48	98.77	97.19
12	0.76	102.42	-13.35	58.02	98.79	97.79
13	0.70	105.04	-21.67	25.47	98.84	95.52
14	0.65	100.58	-7.29	75.14	98.85	97.35
15	0.45	94.53	-9.49	27.73	98.81	93.88
16	0.89	98.11	-3.60	53.49	98.06	95.81
17	0.85	106.31	-9.72	43.54	98.29	95.94
18	0.78	101.74	-9.87	49.82	98.72	95.72
19	0.24	99.13	-2.02	47.46	98.94	98.64
20	0.77	100.94	-7.43	38.72	98.77	97.41
21	0.61	102.00	-13.26	41.16	96.28	89.62
22	0.51	99.19	-8.62	72.32	98.74	96.74
23	0.77	104.01	-14.21	25.10	98.93	98.05
24	0.99	104.21	-18.68	50.34	98.63	97.97
25	0.85	100.37	-5.43	92.48	98.91	98.68
26	0.85	101.16	-7.38	63.53	98.88	98.46
27	0.94	103.50	-19.57	40.70	98.43	96.80
28	0.59	94.07	-5.78	31.83	98.35	92.47

Table S3: Comparison of PAE scores across all investigated systems.

protein	R	Intercept	Slope	PAE_{median}
1	0.92	2.16	2.47	10.5
2	0.89	4.41	1.94	14.3
3	0.85	3.18	2.21	8.8
4	0.59	1.64	3.73	4.2
5	0.55	4.36	0.81	6.5
6	0.83	2.07	2.29	5.8
7	0.83	4.66	4.05	10.0
8	0.41	6.27	2.42	9.5
9	0.65	2.25	0.38	3.3
10	0.84	1.51	2.49	3.2
11	0.56	0.54	2.21	2.6
12	0.46	1.02	2.77	2.3
13	0.58	0.71	4.22	3.7
14	0.35	2.16	0.72	2.6
15	0.51	4.25	1.61	4.2
16	0.65	2.45	0.94	3.2
17	0.76	1.49	1.15	5.1
18	0.45	2.4	0.94	3.2
19	0.25	1.68	0.68	1.9
20	0.78	1.78	1.31	2.7
21	0.88	1.57	2.65	13.2
22	0.66	2.59	4.83	7.6
23	0.46	2.51	0.43	3.1
24	0.79	0.1	4.83	1.9
25	0.58	0.93	2.49	1.9
26	0.72	1.02	2.03	2.0
27	0.81	2.22	1.7	7.2
28	0.56	5.34	0.58	9.2

Table S4: Two-dimensional Kullback-Leibler divergence values of the first two principal component distributions of aMD against cgMD simulations with different ENMs. The cgMD trajectories are projected on the aMD-derived PCs. A lower value corresponds to a higher similarity between both distributions.

protein	Residues	ENM	AF-ENM	AF-ENM (const)	AF-ENM x 2 *	AF-ENM +6% P-W interaction	AF-ENM +6% P-W interaction (const)
24	1 - 70	1.1	1.8	0.8	0.7		
24	1 - 76 (all)	0.8	1.1	1.2	1.2		
25	1 - 128 (all)	4.5	1.4	1.6	1.3		
26	5 - 228	12.1	11.4	11.7	12.0		
26	1 - 238 (all)	11.0	4.8	5.3	5.2		
27	1 - 152 (all)	9.4	5.9	5.9		6.1	6.4
28	1 - 210 (NTD)	11.9	1.2	1.3		1.2	1.3
28	275-524 (MD)	9.0	4.8	4.8		4.0	5.1
28	1 - 524 (all)	10.5	4.4	4.8		4.6	4.1

* AF-ENM with twice as strong force constants

Table S5: Correlation between experimental factors of C_α atoms and the RMSF from single-domain aMD and cgMD simulations. The B-factors are taken from PDB ID: 1UBQ (protein 24, ubiquitin), 1DPX (protein 25, lysozyme C), 5B61 (protein 26, green fluorescent protein).

protein	Residues	aMD	ENM	AF-ENM	AF-ENM (const)	AF-ENM x 2
24	1 – 76 (all)	0.71	0.52	0.61	0.56	0.57
25	1 - 128 (all)	0.80	0.80	0.83	0.86	0.86
26	8 - 228 (all)	0.52	0.12	0.15	0.10	0.09
